# TLR-9 Contributes to the Antiviral Innate Immune Sensing of Rodent Parvoviruses MVMp and H-1PV by Normal Human Immune Cells

**DOI:** 10.1371/journal.pone.0055086

**Published:** 2013-01-29

**Authors:** Zahari Raykov, Svitlana P. Grekova, Rita Hörlein, Barbara Leuchs, Thomas Giese, Nathalia A. Giese, Jean Rommelaere, Rainer Zawatzky, Laurent Daeffler

**Affiliations:** 1 Infection and Cancer Program, Division F010 and Institut National de la Santé et de la Recherche Médicale INSERM U701, German Cancer Research Center, Heidelberg, Germany; 2 Infection and Cancer Program, Division F030 Department Viral Transformation Mechanisms, German Cancer Research Center, Heidelberg, Germany; 3 Institute of Immunology, University Hospital Heidelberg, Heidelberg, Germany; 4 Department of General, Visceral and Transplantation Surgery, University Hospital Heidelberg, Heidelberg, Germany; University of Birmingham, United Kingdom

## Abstract

The oncotropism of Minute Virus of Mice (MVMp) is partially related to the stimulation of an antiviral response mediated by type-I interferons (IFNs) in normal but not in transformed mouse cells. The present work was undertaken to assess whether the oncotropism displayed against human cells by MVMp and its rat homolog H-1PV also depends on antiviral mechanisms and to identify the pattern recognition receptor (PRR) involved. Despite their low proliferation rate which represents a drawback for parvovirus multiplication, we used human peripheral blood mononuclear cells (hPBMCs) as normal model specifically because all known PRRs are functional in this mixed cell population and moreover because some of its subsets are among the main IFN producers upon infections in mammals. Human transformed models consisted in lines and tumor cells more or less permissive to both parvoviruses. Our results show that irrespective of their permissiveness, transformed cells do not produce IFNs nor develop an antiviral response upon parvovirus infection. However, MVMp- or H-1PV-infected hPBMCs trigger such defense mechanisms despite an absence of parvovirus replication and protein expression, pointing to the viral genome as the activating element. Substantial reduction of an inhibitory oligodeoxynucleotide (iODN) of the latter IFN production identified TLR-9 as a potential PRR for parvoviruses in hPBMCs. However, neither the iODN treatment nor an antibody-induced neutralization of the IFN-triggered effects restored parvovirus multiplication in these cells as expected by their weak proliferation in culture. Finally, given that a TLR-9 activation could also not be observed in parvovirus-infected human lines reported to be endowed with a functional TLR-9 pathway (Namalwa, Raji, and HEK293-TLR9^+/+^), our data suggest that transformed human cells do not sense MVMp or H-1PV either because of an absence of PRR expression or an intrinsic, or virus-driven defect in the endosomal sensing of the parvovirus genomes by TLR-9.

## Introduction

Rodent parvoviruses MVMp (mouse) and H-1PV (rat) are small, non-enveloped, single-stranded (ss) DNA viruses that replicate during the S-phase of the cell cycle within the host nucleus [1]. Both viruses share around 86% DNA sequence homology. The viral genome contains two overlapping open-reading frames encoding nonstructural regulatory polypeptides (NS1 and NS2) and capsid proteins (VP1 and VP2). Expression of the former polypeptides is regulated by the early P4 promoter whereas the VP expression is controlled by the NS1-inducible P38 promoter [2]. Both viruses belong to the genus Parvovirus of the *Parvoviridae* family. Adeno-Associated Viruses (AAVs) represent additional members of this family, however those belong to another genus, the Dependovirus, and their replication requires the helper functions of a co-infecting DNA virus, like Adenovirus or Herpes simplex virus [3], [4]. MVMp and H-1PV are, in contrast to AAVs, endowed with oncotropic and oncolytic properties making them attractive for the development of alternative anticancer therapies [5], [6], while AAVs are classically used as vectors for gene therapy purposes [7].

The oncotropism of several natural or engineered lytic viruses like Newcastle Disease Virus (NDV), Vesicular Stomatitis Virus (VSV), Myxoma Virus (MYXV), Herpes Simplex Virus-I (HSV-I), Measles Virus (MV) or Adenoviruses is at least to some extend related to defects affecting the antiviral innate immune machinery of transformed but not of normal host cells. Indeed, in contrast to normal cells that can develop antiviral defense mechanisms against viruses, tumor cells are often devoid of such protections [8], [9]. Thus, transformed cells offer a suitable environment for the lytic multiplication of these agents allowing them to exert oncolytic and oncosuppressive effects.

In normal cells, viruses stimulate an immune reaction through the activation of an antiviral innate immune response representing the first line of defense an organism can develop against an invader. This process is initiated upon recognition of specific viral elements, often consisting in nucleic acids, termed Pathogen-Associated Molecular Patterns (PAMPs) by cellular sensors called Pattern Recognition Receptors (PRRs) [10]. Three major families of viral sensors are identified so far in mammals, membrane-bound Toll-like receptors (TLRs) in particular TLR-3, -7, -8 and -9 mainly expressed in endosomes [11], [12], [13], cytosolic RIG-I-like receptors (RLRs) RIG-I, MDA5 and LGP2 [14], and NOD-like receptors (NLRs) [15]. TLRs and NLRs are mainly functional in immune cells like dendritic cells (DCs), macrophages and B cells [16], [17] whereas RLRs can exert their antiviral activity in a large variety of cell types [18]. The interaction between a PRR and a PAMP stimulates a cascade of downstream signaling pathways eventually leading to the activation of specific transcription factors like NFκB and interferon-regulatory factors (IRF)-3 and/or -7, which induce the transcription of genes coding for antiviral cytokines, the type-I interferons (IFN-αs and IFN-β) [19]. Released antiviral cytokines interact in both a paracrine and an autocrine manner with specific cell surface receptors leading to the stimulation of the IFN-signaling (Jak/STAT)-pathway [20] characterized among others by the phosphorylation, hetero-dimerization and nuclear translocation of the transcription factors STAT_1_ and STAT_2_. In the nucleus, the STAT complex induces the transcription of interferon stimulated genes (ISGs) like Mx1, OAS, PKR, etc…, that contribute directly or indirectly to the establishment of an antiviral state in both infected as well as non-infected cells [21]. However, viruses have also developed species and/or cellular specific escape mechanisms in order to circumvent the antiviral machinery of their host [22], [23].

*Parvoviridae* were considered for a long time to be, in contrast to many other viruses, poor activators of innate or adaptive immune reactions [24], [25], [26], [27], [28]. This point of view changed however in the last years. Indeed, we showed that the oncotropism of MVMp is at least in part regulated by antiviral innate immune mechanisms activated upon infection in primary but not in transformed mouse cells [29]. In addition, H-1PV and MVMp were both reported to exert oncosuppressive effects in tumor-bearing animals through the activation of an anticancer immune response [30], [31], an effect which could for instance be attributed in part to the triggering of an IFN production by normal infected cells [32]. Finally, another autonomously-replicating rat parvovirus, the Kilham Rat Virus (KRV), was recently shown to induce diabetes in rats upon activation of an immune response [33].

Since autonomously-replicating rodent parvoviruses attract more and more attention because of their anticancer potential against human tumors, it is important to define the role played by the immune system in their oncotropism and oncosuppressive activities. Unfortunately, MVMp as well as H-1PV are not naturally hosted by human cells suggesting that our conclusions regarding the involvement of antiviral mechanisms in the MVMp oncotropism in mouse cells [29] cannot be directly extrapolated to human models [29]. Moreover, no information are so far available regarding the ability of antiviral factors to sense H-1PV, although this virus is presently evaluated in a clinical trial for its anticancer effects against glioblastoma multiforme [6].

Based on all these considerations, we undertook experiments to assess whether both rodent parvoviruses can evoke an antiviral innate immune response in human cells through the stimulation of a type-I IFN production. Our present work provides evidences indicating that these antiviral effects are indeed triggered by MVMp and H-1PV in freshly isolated peripheral blood mononuclear cells (hPBMCs) although both infections proved to be fully defective. These findings suggest that antiviral mechanisms could contribute to both the oncotropism as well as the antitumor activities of rodent parvoviruses. We also show that the cytokine production depends mostly on the activation of the DNA sensor TLR-9, pointing to the parvovirus genome as the viral element recognized as a pathogen-associated molecular pattern (PAMP). In contrast, none of the human transformed or tumor cell lines tested, although some are reported to be endowed with a functional TLR-9 pathway, showed signs of activation of this PRR upon parvovirus infections, suggesting that in neoplastic cells factors and/or mechanisms hamper the TLR-9 ability to sense parvovirus genomes.

## Materials and Methods

### Materials

The rabbit antiserum αSP8 raised against the NS1 protein of MVMp and H-1PV was described previously [34], [35]. The mouse monoclonal anti-STAT_1_ and anti-PKR, as well as the rabbit polyclonal anti-STAT_2_ antibody and the goat polyclonal anti-IRF-3 antibody were all from Santa Cruz Biotechnology (Heidelberg, Germany). The polyclonal rabbit antibody directed against the phospho(Tyr^701^)- α and β isoforms of STAT_1_ were obtained from Cell Signalling (Frankfurt, Germany). The polyclonal rabbit antibody specific for phospho(Tyr^689^)-STAT_2_ was from Millipore (Schwalbach/Ts, Germany). The mouse monoclonal antibody directed against Actin was from MP Biomedicals (Heidelberg, Germany). The synthetic double-stranded RNA (dsRNA) Poly(I:C) was from GE Healthcare Europe (Freiburg, Germany). For transfection, Lipofectamine 2000 from Invitrogen (Karlsruhe, Germany) was used. Recombinant human interferon beta (rhIFN-β) and the ELISA kits for detection of human IFN-αs and -β were both obtained from R&D Systems (Wiesbaden, Germany). Neutralizing antibodies against human IFN-αs, -β and their receptor (IFNAR chain 2) were obtained from R&D system (Wiesbaden, Germany). The TLR-9 agonist ODN 2395 as well as the antagonist (ODN TTAGGG) and its inactive derivative ODN TTAGGG Control, were all obtained from Invivogen (Toulouse, France). Neuraminidase (from *Clostridium Perfringens* type V) was from Sigma-Aldrich (Taufkirchen, Germany).

### Cell cultures

Blood samples obtained from healthy individuals were collected by the blood bank and peripheral blood mononuclear cells (PBMCs) were isolated by differential centrifugation over Histopaque (Sigma, Taufkirchen, Germany), washed and cultured in RPMI with 10% heat-inactivated fetal calf serum (FCS) supplemented with 100 units/ml penicillin and 100 µg/ml streptomycin. Human, SV40-transformed NB324K cells [36], were maintained in Minimum Essential Medium (MEM) supplemented with 5% heat-inactivated FCS, 2 mM L-glutamine, 100 units/ml penicillin and 100 µg/ml streptomycin. HEK293, HEK293T, HEK-Blue™-hTLR-9 (InvivoGen, Toulouse, France), and Hela cells were all grown in Dulbecco's Modified Eagle's Medium (DMEM) containing 10% FCS and appropriate antibiotics. The human Burkitt-lymphoma-derived B cell line Namalwa obtained from the ATCC collection was maintained in Roswell Park Memorial Institute (RPMI) 1640 culture medium supplemented with 10% heat-inactivated FCS and 100 units/ml penicillin and 100 µg/ml streptomycin.

### Virus productions

Primary stocks of wild-type MVMp (mouse) and H-1PV (rat) parvoviruses were produced at the virus production Unit of the DKFZ, by calcium phosphate transfection of HEK293T cells with the pdBMVp or pSR19 infectious molecular clones of MVMp and H-1PV respectively, previously described [37]. Cells were harvested 3 days post-transfection, and viruses were collected by repeated cycles of freezing and thawing in vTE (50 mM Tris-HCl [pH 8.3], 0.5 mM EDTA). Crude cell extracts were then used to re-infect once human NB324K cells for a single further amplification of the stock. After subjecting infected NB324K cells to another series of freeze-thaw cycles in vTE buffer and the resulting supernatant to a Benzonase treatment to destroy non encapsidated viral nucleic acids, virus stocks were purified by non-ionic iodixanol gradient centrifugation [38]. Viral stocks were titered by plaque assays on human NB324K cell monolayers infected with serial dilutions of virus, and expressed as PFUs (Plaque Forming Units)/ml. The multiplicity of infection (MOI) is expressed in PFUs/cell. The contamination of virus stocks with endotoxins tested lower than 2.5 EU/ml. The Lentogenic NDV *Ulster 2C virus* was propagated in embryonated chicken eggs, harvested from the allantoic fluid, purified by ultracentrifugation as described [39] and cryopreserved in aliquots at −80°C. The virus was quantified by a hemagglutination assay. One hemagglutination unit (HU) is defined as the smallest virus concentration leading to visible sheep erythrocyte agglutination.

### Cell transfection with Poly(I:C)

Transfections of NB324K, HEK293, HEK293T, and Hela cells were carried out using Lipofecamine 2000 according to the manufacturer's instructions. Cells were transiently transfected with synthetic dsRNA Poly(I:C) at a final concentration of 2 µg/ml for the times indicated, before being processed for further analysis.

### Cell infection

Cell monolayers (HEK293, HEK-Blue™-hTLR-9, HEK293T, Hela and NB324K) were infected with viruses at the MOI indicated in each figure using serum-free media. After 1 hour, complete medium was added onto the cells. They were then harvested following infection at times indicated in each figure. For cell suspensions (hPBMCs and Namalwa), viruses were directly applied, at the MOI indicated in each figure, into the cell suspension using a small volume of complete media.

### Real time quantitative polymerase chain reactions and semi-quantitative RT-PCRs

For qRT-PCR all reagents and equipment for mRNA/cDNA preparation were supplied by Roche Applied Science (Mannheim, Germany). Messenger RNAs (mRNAs) were prepared by automated isolation using the MagNA pure LC instrument and isolation kit I. cDNA was prepared using the first-strand cDNA synthesis kit for RT-PCR according to the manufacturer's instructions. [40].

For RT-PCRs, total RNA was extracted as previously described [29]. Briefly, Total RNAs of mock-treated, parvovirus- or NDV-infected, and/or PolyI:C transfected cells were isolated using an RNeasy mini kit (QIAGEN, Hilden, Germany) according to the manufacturer's instructions. One µg of total RNA was then digested with 1 unit of DNase I (Promega, Mannheim, Germany) at 37°C for 25 min to remove genomic DNA contamination before being processed for reverse transcription (RT) using oligo(dT) primers and Reverse Transcriptase from M-MLV (Promega, Mannheim, Germany). For each cDNA sample generated in this way, a control was produced using a RT mixture in which reverse transcriptase was omitted in order to detect a potential contamination of the cDNA sample with residual genomic DNA. Ten percent of the resulting cDNA samples were then used as a template for PCR using Taq DNA polymerase (Invitrogen, Karlsruhe, Germany) and the following specific sets of primers (MWG Biotech, Ebersberg, Germany):

for **18S rRNA**, forward primer 5′CGGCTACCACATCCAAGGAA3′ and reverse primer 5′GCTGGAATTACCGCGGCT3′; for **IFN-β**, forward primer 5′ATTGCCTCAAGGACAGGATG3′ and reverse primer 5′AGCCAGGAGGTTCTCAACAA3′; for **IFN-αs**, forward primer 5′TGATGGCAACCAGTTCCAGAAGGCTCAAG3′ and reverse primer 5′ACAACCTCCAGGCACAAGGGCTGTATTT3′ [41]; for **IFN-α2**, forward primer 5′AAATACAGCCCTTGTGCCTGG3′ and reverse primer 5′GGTGAGCTGGCATACGAATCA3′ [42]; for **2′-5′-OAS** (2′-5′-oligoadenylate synthetase), forward primer 5′GTGCTCCTCCGCTGTAAGAC3′ and reverse primer 5′ACAGAACCTCCCAACAGGTG3′; for **H-1PV**, forward primer 5′TGAGCTGGCCCTGACGCTGAA3′ and reverse primer 5′TCGTAGGCTTCGTCGTGTTCT3′ as previously described [43], and for **MVMp**, forward primer 5′ACGCTCACCATTCACGACACCGAAA3′ and reverse primer 5′ATCATAGGCCTCGTCGTGCTCTTTG3′. PCR products were then analyzed by electrophoresis through 2% agarose gels.

### Viral DNA extraction and Southern blot analysis

Viral DNA intermediates were isolated using a modified Hirt extraction method, as previously described [37]. Briefly, medium from mock-treated or MVMp-infected cultures was discarded at the time points indicated in Figure legends, and cells were scraped in PBS and pelleted by centrifugation at 500 *g* for 5 min at room temperature (RT). Cell pellets were resuspended in a 1∶1 mixture (v/v) of vTE buffer and 2× Hirt buffer (20 mM Tris [pH 7.4], 20 mM EDTA, 1.2% SDS), followed by proteinase K digestion (400 µg/ml) for 18 h at 46°C. Cellular genomic DNA was sheared by five passages through 0.5 and then 0.4 mm needles. DNA samples (2 µg) were fractionated by electrophoresis on a 0.8% agarose gel. After denaturation, the DNA was immobilized onto a nylon Hybond N^+^ membrane (Amersham Biosciences). Viral DNA intermediates were detected, after denaturation and neutralization, by hybridization of the membrane with a mixture of ^32^P-labeled DNA probes corresponding to the *Eco*RV (nt 385)-*Eco*RI (nt 1084) and the *Hind*III (nt 2647)-*Hind*III (nt 7531) fragments of the MVMp and H-1PV NS genes, respectively.

### SDS-PAGE and Western blotting

At the indicated time points, mock-treated or infected/transfected/stimulated cells were scraped in PBS and centrifuged at 500 *g* for 5 min at room temperature. Cell pellets were resuspended in modified radio-immunoprecipitation assay (RIPA) buffer (50 mM Tris-HCl [pH 7.4], 150 mM NaCl, 1 mM EDTA, 1% NP-40, 0.25% Na-deoxycholate, protease inhibitor cocktail [Roche Diagnostics] and phosphatase inhibitors: 20 mM NaF, 5 mM *ß*-glycophosphate, 5 mM *p*-nitrophenyl phosphate, 5 mM sodium molybdate, 1 mM sodium orthovanadate, 5 mM sodium phosphate) and stored on ice for 30 min. Samples were centrifuged at 20,000 *g* for 15 min at 4°C, and the protein concentration in the supernatants determined using the Pierce BCA protein assay kit according to the manufacturer's instructions (Pierce Biotechnology, Rockford, U.S.A.). Samples were then boiled for 5 min in Laemmli buffer, fractionated by 8 or 10% SDS-PAGE, and blotted onto nitrocellulose membranes (Schleicher & Schuell, Dassel, Germany). The membranes were then blocked with 1× PBS containing 5% low-fat dry milk and 0.1% Tween-20 for 1 h. For detection of phosphorylated proteins, 1× Tris-buffered saline solution (TBS: 20 mM Tris-HCl [pH 7.6], 137 mM NaCl) containing 0.1% Tween-20 and 2% casein was used as a blocking solution. Incubations with primary antibodies were carried out at 4°C overnight either in 1× PBS containing 5% low-fat dry milk and 0.1% Tween-20, or in 1× TBS supplemented with 0.1% Tween-20 and 5% bovine serum albumin. Individual proteins were identified by means of specific antibodies used at a 1∶2,000 (αSP8-NS1) or 1∶1,000 (others) dilution. Protein-antibody complexes were then visualized with horseradish peroxidase-conjugated anti-rabbit (1∶10,000 dilution) or anti-mouse (1∶5,000 dilution) IgGs (Promega). The immunoreactive total and phosphorylated proteins were detected by ECL (Amersham Biosciences, Freiburg, Germany).

### Detection of type-I IFN production

Secretion of type-I IFNs by virus-infected, pI:C-transfected or ODN-treated cell cultures was determined by Enzyme-Linked Immunosorbent Assay (ELISA). Briefly, culture supernatants of mock-treated or stimulated cells were collected at indicated time points and cleared of cell debris by a brief centrifugation (500 *g* for 5 min). Concentrations of type-I IFNs (α and β) were then quantified in the supernatants using ELISA kits from R&D Systems (Wiesbaden, Germany) following the manufacturer's instructions.

## Results

### Assessment of the intrinsic capacity of four human cell lines to produce and release type-I IFNs and to mount an antiviral response

**Figure 1:**
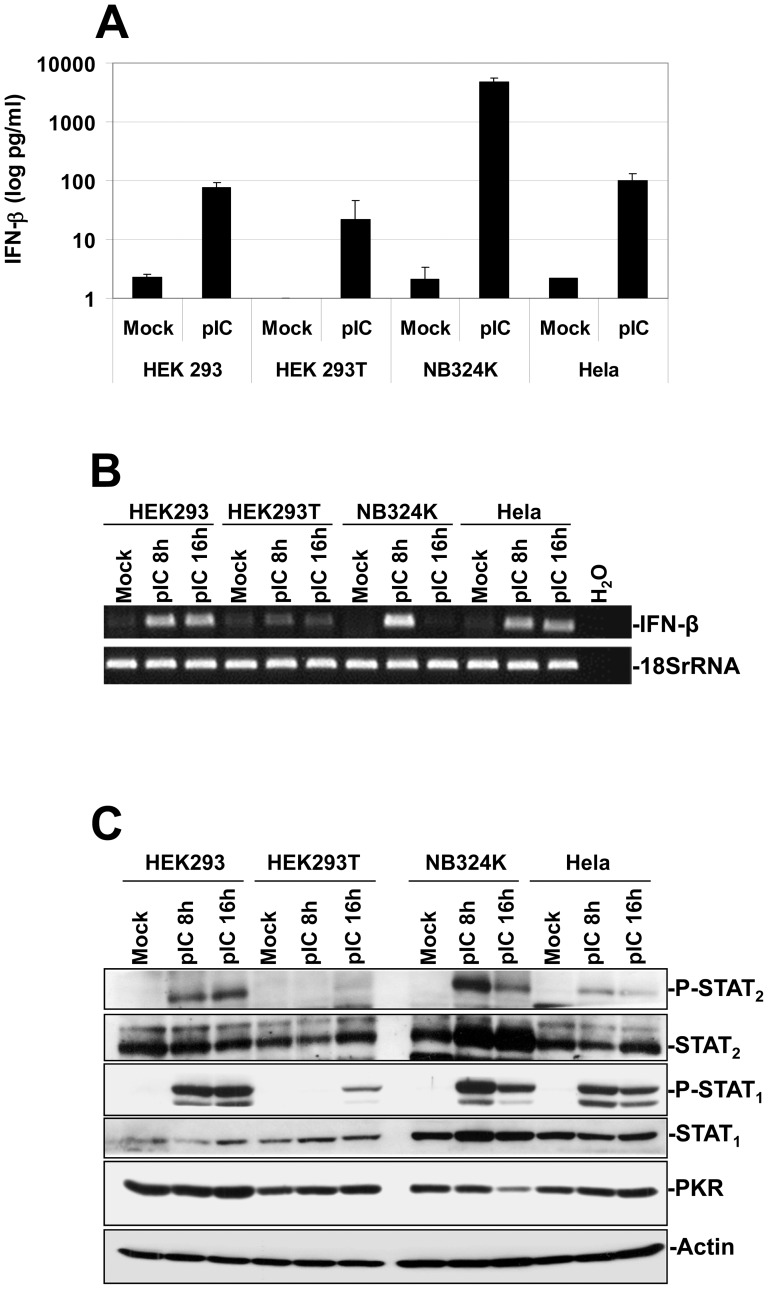
Stimulation of an IFN-mediated antiviral response in human transformed or tumor cells upon Poly(I:C) transfection. (**A**) HEK293, HEK293T, NB324K and Hela cultures were transfected with 2 µg/ml of synthetic dsRNA Poly(I:C) (pI:C) or a 150 mM NaCl solution as control, using lipofectamine 2000 for the period indicated in the figure. The respective culture media were then collected, centrifuged to discard cellular debris, and analyzed by Enzyme-linked Immuno-Sorbent Assay (ELISA) for their content in human IFN-β. Each result is represented as mean+standard deviation of three independent experiments. (**B**) Expression of IFN-α and IFN-β transcripts in mock-treated or pI:C-transfected human cell lines was assessed by RT-PCRs. At the indicated time points, total RNAs were extracted from the respective cultures using the RNeasy kit. One µg of RNA was then treated by DNase I in order to remove potential contaminating DNA and reverse transcribed into cDNA. A fraction of the obtained cDNA (1/10) was then analyzed by PCR for its content in type-I IFN transcripts using specific primer pairs. Transcripts encoding the Human 18S ribosomal protein were used as internal loading controls. Data shown are representative of 3 experiments which gave similar results. (**C**) Assessment of the IFN-signaling (Jak/STAT) pathway activation in HEK293, HEK293T, NB324K and Hela cells upon pI:C transfection. At the time indicated mock-treated or pI:C-transfected (2 µg/ml) cultures were harvested by scraping in PBS and centrifuged. Cell pellets were then re-suspended in complete Ripa buffer supplemented with phosphatase and protease inhibitors. Total proteins were extracted from each sample as described in Materials and Methods. Fifty µg total proteins per sample were then subjected to bipartite 8/10% SDS-PAGE, transferred onto membranes, and probed with antibodies specific for phosphorylated and total isoforms of STAT_1_ and STAT_2_ as well as with an antibody specific to PKR. Actin was used as an internal loading control. Each presented blot is representative of 3 additional which gave similar results.

Before assessing whether human transformed/tumor cells produce type-I IFNs and develop an antiviral response upon MVMp or H-1PV infections, we first characterized the ability of some lines known to be more (NB324K and HEK293T) or less (HEK293 and Hela) permissive to these viruses, to produce and release IFN-β upon their exposure to classical activators of antiviral defense mechanisms like Poly(I:C), a synthetic dsRNA, (pI:C) [44], or the avian paramyxovirus Newcastle Disease Virus (NDV, *Ulster* strain) [39], [45], [46]. The production of this antiviral cytokine represents indeed a hallmark of activation of an antiviral response in non-immune cells. Using pI:C, we show Figure 1A, that while mock-treated transformed cells did not release IFN-β as assessed by Enzyme-Linked Immuno-Sorbent Assays (ELISA), a strong production of the latter cytokine, reaching 4832±644 pg/ml, was detected in pI:C transfected NB324K cultures. In contrast, only low amounts of the cytoine (∼100 pg/ml or less) were measured in the culture supernatant of the other pI:C-transfected lines. Increasing the quantity of transfected synthetic dsRNA up to 20 µg/ml did however not increase the amount of released cytokine in any of the lines tested (data not shown). Interestingly, similar amounts of IFN-β as those measured upon pI:C transfection were released by each cell line upon their infection with NDV at an MOI of 6 HU/1×10^6^ cells (data not shown)

We then assessed whether the more sensitive technology RT-PCR could allow a better detection of the above described IFN-β productions. Using this method, we indeed observed that with the exception of HEK293T cells in which only a weak increase in IFN-β transcription was observed upon pI:C transfection, all the other lines showed an obvious up-regulation in the expression of mRNAs coding for the antiviral cytokine compared to mock-treated cultures (Figure 1B).

Taken together, these findings indicated that apart from NB324K cells that seemed to possess at least fully functional RLR-dependent/IFN-producing pathways, all the other lines appeared to be rather defective for antiviral cytokine production although some synthesis can be evidenced using the RT-PCR technology.

We then assessed using the synthetic dsRNA and Western blot approaches the integrity and functionality of the IFN-signaling (Jak/STAT) pathway of the transformed cell lines. As shown in Figure 1C, we indeed detected upon pI:C transfection early hallmarks of activation of the Jak/STAT-pathway in NB324K, HEK293, and to a lesser extent in Hela cells as demonstrated by the phosphorylation of both STAT_1_ and STAT_2_ transcription factors. It is worth noting that despite a weak release of IFN-β triggered by the dsRNA transfection in HEK293 and Hela cells (Figure 1), these quantities of antiviral cytokine seemed nevertheless sufficient to induce a quite strong phosphorylation of both transcription factors. In contrast, transfected HEK293T monolayers showed only weak STAT_1_ phosphorylation and no activation/phosphorylation of STAT_2_. We also investigated more downstream hallmarks of activation of the latter pathway in these lines by assessing the expression level of the IFN-stimulated genes (ISGs) STAT_1_, STAT_2_ and PKR. We could however not detect, except in pI:C-challenged NB324K cells in which an up-regulation in STAT_1_ and STAT_2_ expression was detected, any obvious enhancement in the steady-state levels of the latter ISGs compared to control cells (Figure 1C).

Altogether these data suggested that the four transformed cell lines tested do not possess a fully functional IFN-signaling pathway, however the amount of antiviral cytokines released at least upon pI:C transfection seemed sufficient to activate to a detectable level early events of the the latter pathway. This assumption was further confirmed by our observation of a complete absence of inhibitory effect triggered by a pre-treatment of the lines with recombinant human IFN-β (rhIFN-β, 500 IU/ml) against the MVMp or H-1PV replication (Figure S1). Thus, apart from NB324K cells, all the other lines appeared endowed with severe intrinsic deficiencies affecting both their sensitivity to type-I IFNs as well as their capacity to produce these antiviral cytokines. However, using RT-PCR and Western blot technologies we show that it is possible to detect some weak IFN productions and IFN-triggered effects in at least three of the four lines.

### Assessment of an antiviral response triggered by MVMp or H-1PV in infected human cell lines

**Figure 2:**
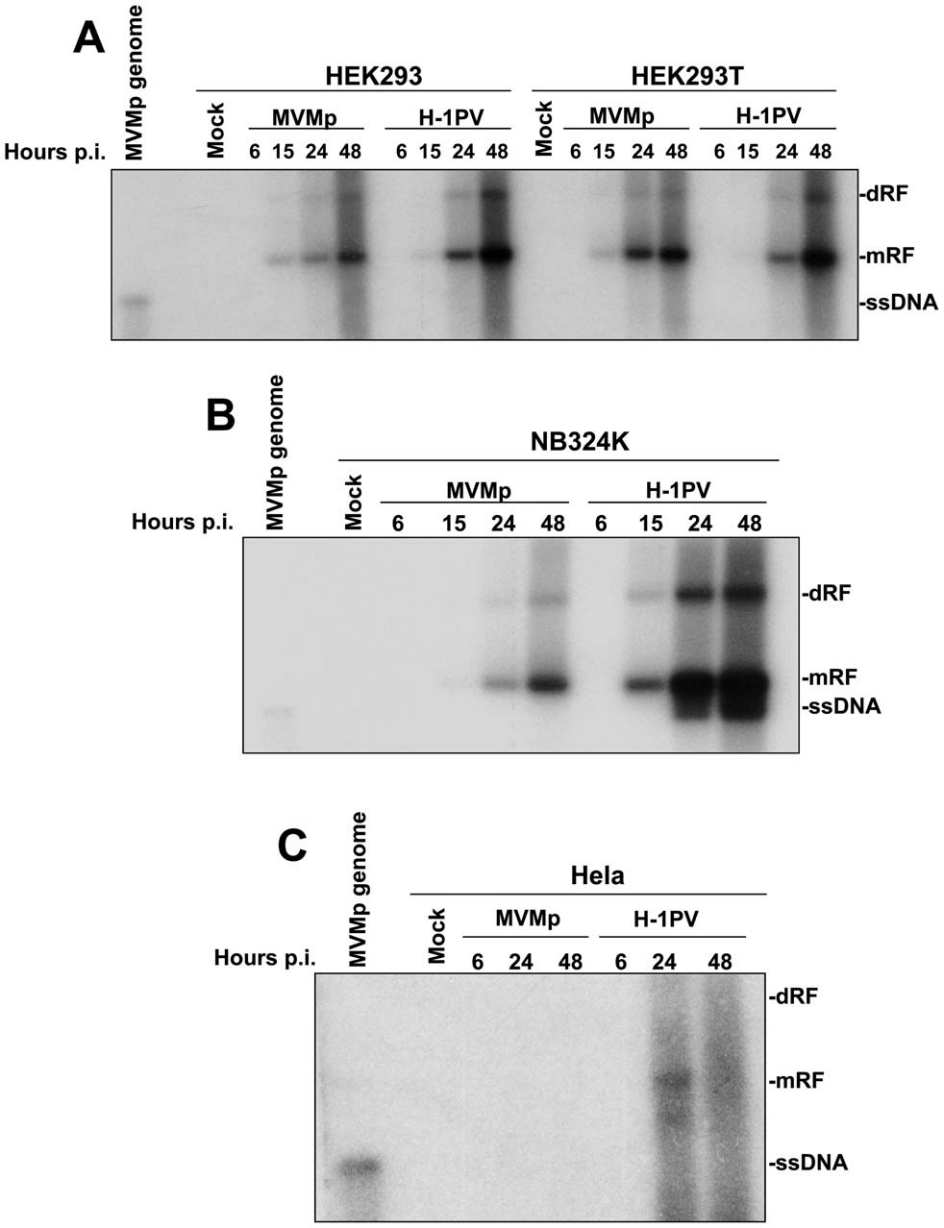
Replication of MVMp and H-1PV in human transformed or tumor cell lines. HEK293 and HEK293T (A), NB324K (B) and Hela (C) cells were mock-treated or infected with MVMp or H-1PV at 5 PFUs/cell. At the indicated time post-infection (p.i.) cells were harvested and DNA was extracted according to a modified Hirt procedure (see Materials and Methods). Samples were then digested with proteinase K and 2 µg of DNA from each sample was then subjected to eletrophoresis through a 0.8% agarose gel and further transferred by capillarity on a Hybond-N membrane. Expression of DNA intermediates was investigated using a mixture of radiolabeled DNA probes corresponding to the E*co* RI-E*co* RV and H*ind* III-H*ind* III fragments of the viral NS genes from MVMp and H-1PV, respectively. Assessment of the migration of MVMp isolated genomes (0.08 µg) was used in each blot as migration control of the different DNA intermediates; mRF, monomeric replicative form; dRF, dimmer replicative form; ssDNA, single-stranded genome. The blots shown are representative of 3 experiments which all gave similar results.

Taking into account the previous results, we then determined whether MVMp and H-1PV trigger an IFN-mediated antiviral response in these transformed/tumor cell lines. We first compared by Southern blot experiments the ability of both viruses to time-dependently replicate in these cultures. We noticed (Figure 2) that with the exception of Hela cells in which H-1PV showed a weak and MVMp even a total absence of replication (Figure 2C), both parvoviruses produced in a time-dependent manner increasing amounts of viral DNA intermediates (dimeric replicative form (dRF), and monomeric replicative form (mRF)) in HEK293, HEK293T and NB324K cells (Figure 2A and B), features typically characterizing the replication of rodent parvoviruses in permissive hosts. Interestingly, ssDNA progeny genomes were only detected in H-1PV-infected NB324K cells. Their absence in the other infected cells, apart from Hela cells, most likely reflects a rapid egress of the produced virions outside of the cells rather than a lack of virus production. However, this explanation has yet to be experimentally demonstrated. Nevertheless, the fact that both HEK293T and NB324K cells are classically used in laboratories as MVMp- or H-1PV-producing cell lines further demonstrates that at least these two lines can indeed produce both parvoviruses efficiently. Noteworthy, although MVMp and H-1PV were applied at the same MOI, replication of the latter virus seemed each time more efficient than that of the former.

**Figure 3:**
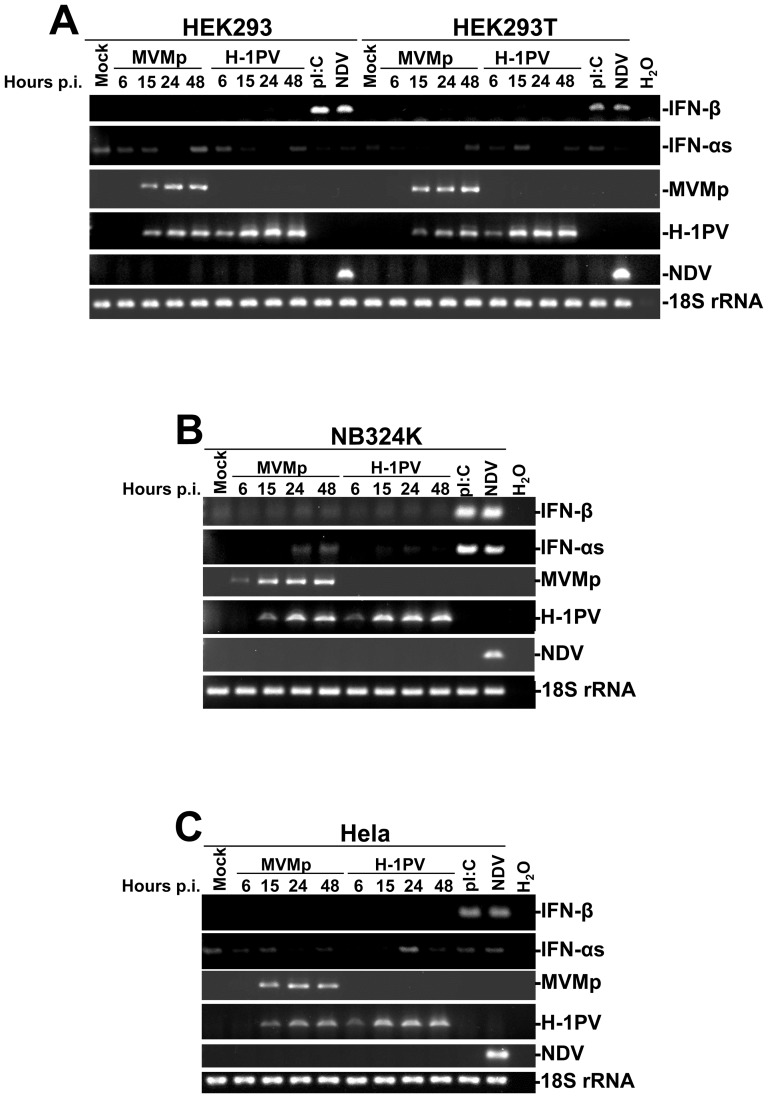
Time-dependent transcriptional up-regulation of type-I IFN encoding mRNAs in MVMp- or H-1PV-infected human cell lines. (**A**) HEK293 and HEK293T, (**B**) NB324K and (**C**) Hela cells were mock-treated for 24 hrs, infected with parvoviruses (5 PFUs/cell) for the indicated times, or infected with NDV (6 HU/10^6^ cells) or transfected with pI:C (2 µg/ml) for 15 hrs. At the indicated times, cells were harvested and total RNAs were extracted using the RNeasy kit. One µg was then reverse transcribed into cDNA and 10% of this product was subjected to PCR reactions using sets of primers specific to each indicated cytokine mRNA or viral transcripts. PCR product of 18S ribosomal RNA was used as housekeeping gene to normalize loading. No signal was detected in the samples when omitting the reverse transcriptase. Presented data are representative of 3 experiments which all gave similar results.

We then investigated by RT-PCR experiments the ability of both rodent parvoviruses to stimulate the production of type-I IFNs (α/β) in these lines and used pI:C transfections and NDV infections as positive controls. As presented in Figure 3, transcriptional induction of IFNs was only observed when the lines were challenged with pI:C or NDV but never upon parvovirus infection suggesting that these infectious agents do not evoke an IFN-producing pathway in transformed human cells. Additional data further argued in favor of this interpretation. First, ELISA experiments failed to detect IFN-β molecules in the culture supernatant of parvovirus-infected monolayers (Figure S2) (neither IFN-αs, data not shown) and second, no activation of the IFN-signaling pathway (STAT_1/2_ phosphorylation) was observed by Western blot experiments in the latter samples (Figure S3). Interestingly, while H-1PV replicated better than MVMp in each line (Figure 2), no obvious difference in NS1 protein expression could be noticed (Figure S3A and B) in the respective cultures except for Hela cells where the amount of NS1 produced by MVMp seemed to be lower than that produced by H-1PV (Figure S3C). The latter observation is important since it strongly suggests that the lack of parvovirus replication noticed in Hela cells (Figure 2 and S1) most likely depends on a post-entry inhibitory mechanism rather than on an entry defect, although a weaker infectivity of Hela cells compared to HEK293T, HEK293 or NB324K cells cannot be fully excluded in particular for MVMp.

Altogether, these data indicated that rodent parvoviruses MVMp and H-1PV are unable to stimulate the production (transcription) of type-I IFNs in human transformed/tumor cells.

### Assessment of an antiviral response triggered by MVMp or H-1PV in infected human peripheral blood mononuclear cells (hPBMCs)

The absence of an antiviral response triggered by MVMp and H-1PV in transformed human cells prompted us to investigate whether this situation was also encountered in parvovirus-infected normal human cells. To assess this question we choose human peripheral blood mononuclear cells (hPBMCs) as normal cellular model since (i) they contain the whole repertoire of known PRRs in a functional status, (ii) some of the immune cell types constituting this heterogeneous mixture, in particular B cells and plasmacytoid dendritic cells (pDCs), are known to be the main producers of type-I IFNs in mammals, (iii) they are easily isolated from blood, and finally (iv) they do not require any previous culture steps. For all these reasons hPBMCs represented to our opinion a rather good normal cellular model although their weak proliferation in culture represents most likely an important drawback for the proper fulfillment of the parvovirus life-cycle.

**Figure 4:**
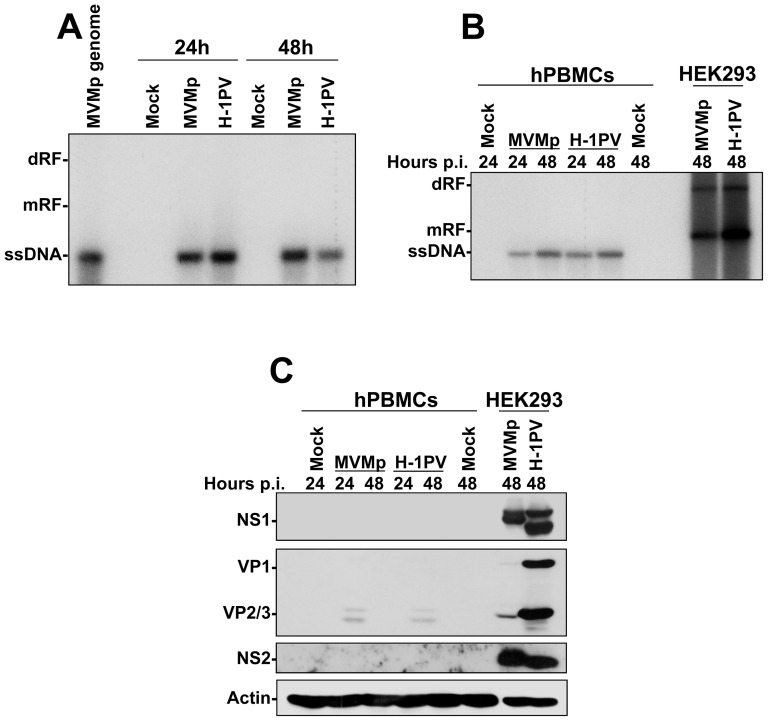
Defective replication of parvoviruses in hPBMCs. (**A**) hPBMCs collected from the blood of healthy donors were distributed into 6-well plates at 1×10^7^ cells/5 ml culture medium/well. They were then mock-treated or infected with MVMp or H-1PV at 10 PFUs/cell and 24 hrs later harvested for Southern blotting as described in Figure 2. The presented blot is representative of 3 additional which gave similar results. (**B**) hPBMCs (1×10^7^ cells/5 ml culture medium/well of a 6-well plate) and HEK293 (1.5×10^6^ cells/10-cm dish) were mock-treated or infected with the indicated parvovirus at 10 and 2 PFUs/cell respectively, and for the indicated period of time. Cultures were then harvested for Southern blotting as described in Figure 2. The presented blot is representative of 2 additional which gave similar results. (**C**) Expression of the parvovirus proteins in infected hPBMCs was examined in the same samples as those already analyzed for parvovirus replication in (**B**). At the time point indicated mock-treated or parvovirus-infected cultures were harvested by scraping in PBS and centrifuged. Cell pellets were then re-suspended in complete Ripa buffer supplemented with phosphatase and protease inhibitors. Total proteins were extracted from each sample as described in Materials and Methods. Fifty µg total proteins per sample were then subjected to 8% SDS-PAGE, transferred onto membranes, and probed with laboratory produced polyclonal antibodies specific for parvovirus NS1, NS2 and capsid polypeptides (Note: The antibody specific to VP proteins was designed to detect H-1PV polypeptides and is therefore less specific for MVMp). Actin was used as an internal loading control. The presented blot is representative of 2 additional which gave similar results.

Therefore, we first determined by Southern blot experiments whether MVMp and H-1PV replicate in hPBMCs. As suspected, only viral ssDNA genomes were detected in infected immune cells arguing indeed for an absence of replication of the latter agents (Figure 4A). These results contrasted strongly with those obtained in infected HEK293 cultures, in which replicative viral DNA intermediates (mRF and dRF) were evidenced demonstrating an efficient parvovirus replication (Figure 4B). Moreover, in contrast to HEK293 cultures, parvovirus-infected hPBMCs did also not express viral non-structural (NS1 and NS2) or capsid (VP1 and VP2) proteins (Figure 4C). Altogether, these data indicated that in contrast to transformed human cells, MVMp and H-1PV fail to establish an infection in freshly isolated hPBMCs.

**Figure 5:**
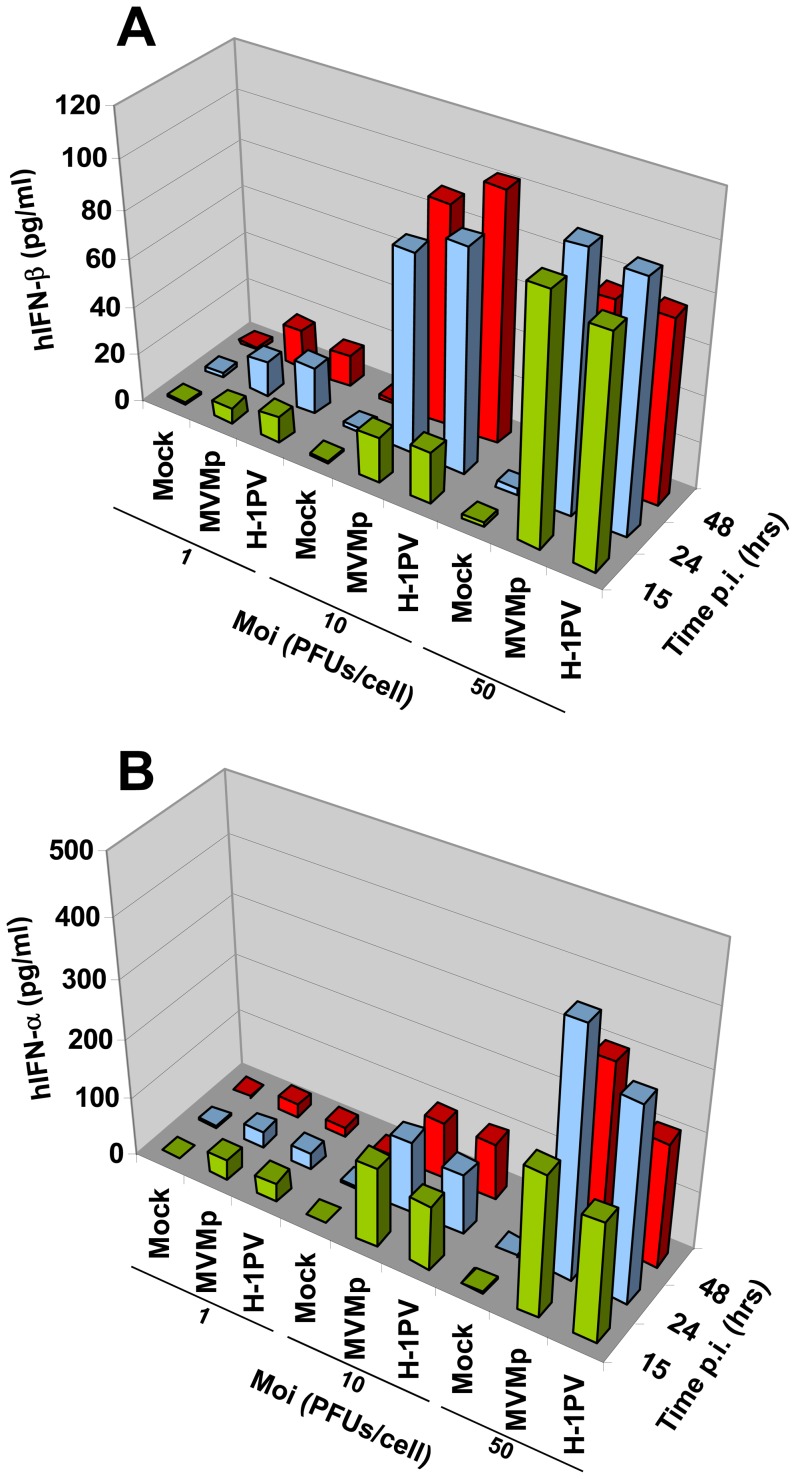
Time- and MOI-dependent production and release of type-I IFNs from parvovirus-infected hPBMCs. hPBMCs collected from the blood of healthy donors were distributed into 24-well plates at 1×10^6^ cells/500 µl culture medium/well. They were then mock-treated or infected with MVMp or H-1PV at 1, 10 or 50 PFUs/cell. After a period of incubation of 15, 24 or 48 hrs media were collected, centrifuged in order to discard cellular debris and analyzed by Enzyme-linked Immuno-Sorbent Assay (ELISA) for their content in IFN-α (**A**) and IFN-β (**B**). Results are expressed as means of three experiments.

Although these immune cells were found not adequate for a proper accomplishment of the parvovirus replication, we nevertheless tested by ELISA experiments in a time- and MOI-dependent manner whether they release type-I IFNs upon MVMp or H-1PV infection. Surprisingly, we indeed detected both α and β IFNs in the culture medium of parvovirus-infected PBMCs but not in that of mock-treated cells (Figure 5). In addition, we also noticed a correlation between the medium concentration of IFNs and an increase in MOI or incubation time although a maximal level of released cytokines seemed to be reached at 50 PFUs/cell and/or 24 hrs p.i.

**Figure 6:**
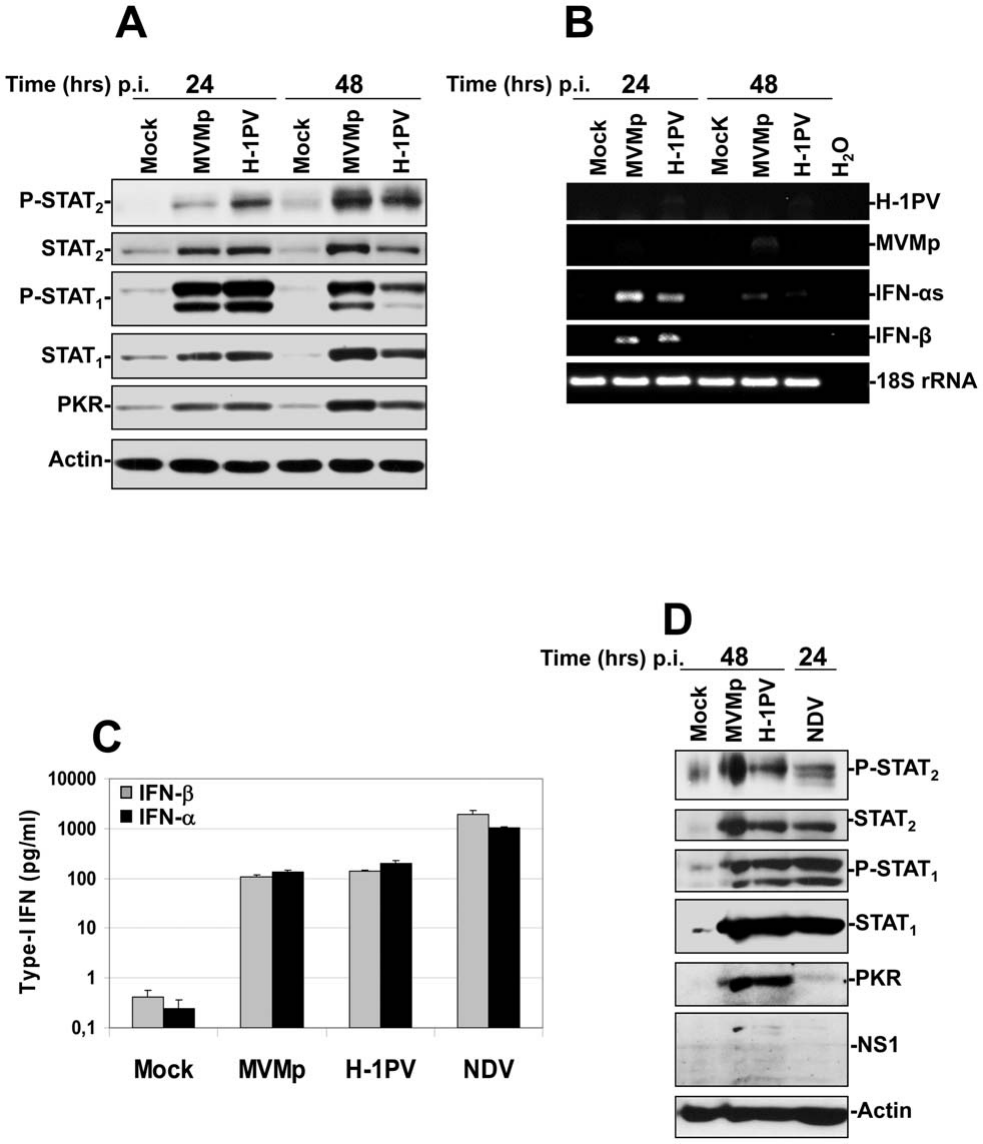
Activation of both IFN-producing and IFN-signaling pathways in hPBMCs upon parvovirus infection and comparison of their intensity to that triggered by NDV. (**A**, **B** and **D**) hPBMCs collected from the blood of healthy donors were distributed into 6-well plates at 1×10^7^ cells/5 ml culture medium/well. They were then mock-treated or infected with MVMp or H-1PV at 20 PFUs/cell or with NDV (6 HU/10^6^ cells). Cultures were harvested in PBS and centrifuged at the time p.i. indicated in each figure. (**A**, **D**) One half of each pellet was resuspended in Ripa buffer supplemented with phosphatase and protease inhibitors in order to perform Western blot experiments whereas (**B**) total RNAs were extracted from the rest of each cell pellet using the RNeasy kit. (**A**, **D**) Total proteins were extracted from each sample as described in Materials and Methods. Seventy µg total proteins per sample were then subjected to 10% SDS-PAGE, transferred onto membranes, and probed with antibodies specific for total and phosphorylated STAT_2_ and STAT_1_ polypeptides as well as for PKR. Actin was used as an internal loading control. Each presented blot is representative of 4 additional which gave similar results. (**B**). One µg of isolated total RNA was then reverse transcribed into cDNA and 10% of this product was subjected to PCR reactions using sets of primers specific to each indicated mRNA. PCR product of 18S ribosomal RNA was used as housekeeping gene to normalize loading. No signal was detected in the samples when omitting the reverse transcriptase. Presented data are representative of 4 experiments which all gave similar results. (**C**) hPBMCs collected from the blood of healthy donors were distributed into 24-well plates at 1×10^6^ cells/500 µl culture medium/well. They were then mock-treated or infected with MVMp, H-1PV (20 PFUs/cell) or NDV (6 HU/10^6^ cells). After a period of incubation of 24 hrs media were collected, centrifuged in order to discard cellular debris and analyzed by Enzyme-linked Immuno-Sorbent Assay (ELISA) for their content in IFN-α and IFN-β. Results are expressed as means+standard deviations of seven independent experiments.

Next, we performed Western blot as well as RT-PCR experiments on parvovirus-infected immune cells to determine whether a classical antiviral response is triggered. As shown in Figure 6A, we observed that upon MVMp or H-1PV infection both transcription factors STAT_1_ and STAT_2_ become activated (phosphorylated) and their expression, as well as that of another ISG the cytosolic protein kinase PKR, increased…Moreover, RT-PCR analysis of the transcription of α and β IFNs in the latter samples revealed also an up-regulation compared to mock-treated cells (Figure 6B). This effect was however fading with time so that at 48 hrs p.i. both types of transcripts became almost undetectable. Taken together, these results indicated that MVMp and H-1PV stimulate a classical antiviral response in human hPBMCs.

We then compared the strength of the antiviral response triggered by parvoviruses in hPBMCs to that evoked by NDV, a very potent inducer of a classical antiviral response in these cells [46]. As expected, we noticed by ELISA experiments that ten times more α and β IFNs were produced by the immune cells upon an NDV infection than upon a rodent parvovirus infection (Figure 6C). However, despite this difference, the downstream stimulation of the IFN-signaling pathway proved to be similar between the parvovirus- and paramyxovirus challenged cells as demonstrated by an equivalent level of STAT_1/2_ phosphorylation and an even higher induction of PKR expression achieved by the former viruses (Figure 6D).

**Figure 7:**
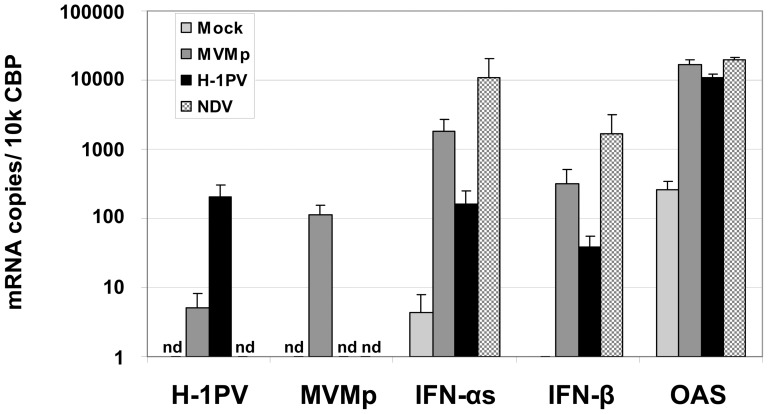
Quantification by real time PCR of the type-I IFN and OAS transcripts produced upon parvovirus infections of hPBMCs. hPBMCs collected from the blood of healthy donors were distributed into 24-well plates at 1×10^6^ cells/500 µl culture medium/well. They were then mock-treated or infected with MVMp, H-1PV (20 PFUs/cell) or NDV (6 HU/10^6^ cells). After a period of incubation of 24 hrs, cells were collected by centrifugation and resuspended in MagNA pure LC solution (Roche) in order to extract in an automated manner mRNAs. cDNAs were produced in the same apparatus using the first-strand cDNA synthesis kit from Roche. QRT-PCRs were then performed using specific sets of primers corresponding to the indicated transcripts. The calculated number of transcripts was normalized to the copy numbers of the housekeeping gene cyclophilin-B. Results are expressed as means+standard deviations of 6 independent experiments.

These results were confirmed in real-time PCR experiments showing that five to ten times higher amounts of α- and β-IFN transcripts were produced in NDV-infected PBMCs (24 hrs p.i.) compared to MVMp- or H-1PV-infected cells (48 hrs p.i.) (Figure 7). However, the transcription level of the ISG 2′–5′ Oligoadenylate Synthetase (OAS),was undistinguishable between the latter samples, as previously observed for PKR (Figure 6). Taken together, these data suggested that under our experimental conditions rodent parvoviruses stimulate a rather weak IFN production compared to NDV in hPBMCs, however strong enough,to trigger a downstream activation of the IFN-signaling (Jak/STAT) pathway similar to that induced by the paramyxovirus.

**Figure 8:**
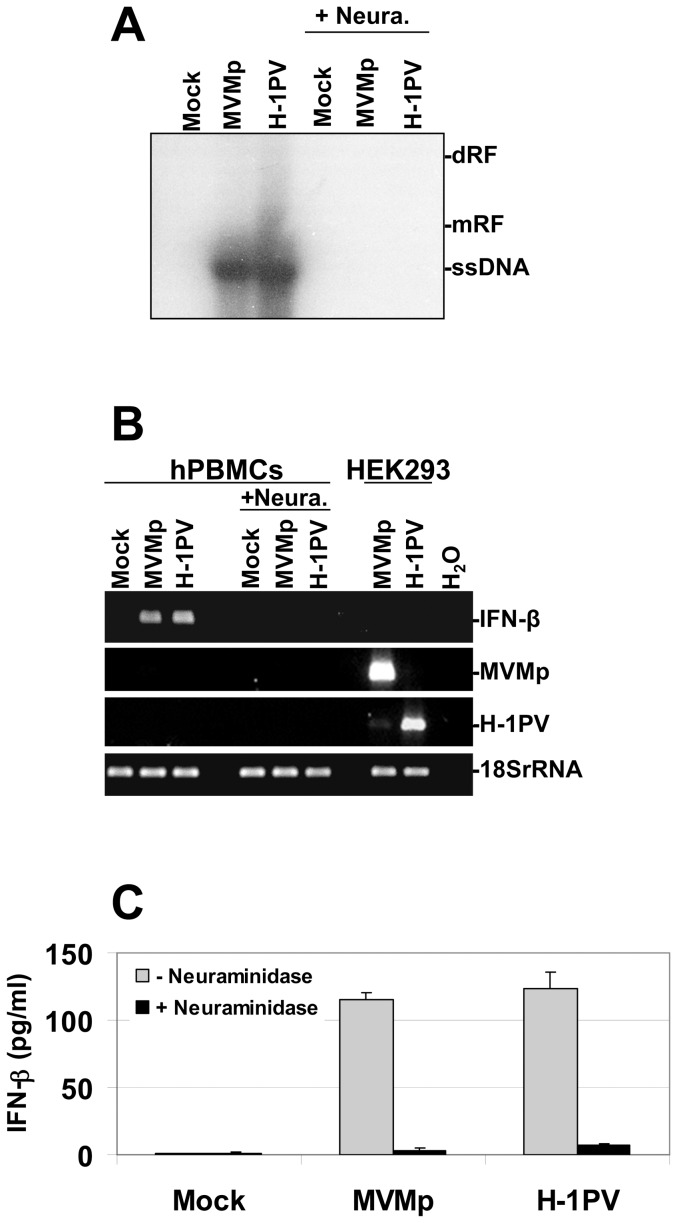
Sensitivity to neuraminidase pre-treatment of the antiviral response triggered by parvoviruses in hPBMCs. (**A** to **C**) hPBMCs collected from the blood of healthy donors were distributed into 6-well plates at 1×10^7^ cells/5 ml culture medium/well. They were then treated or not with neuraminidase at 0.1 U/ml for 15 hrs and then mock-treated or infected with MVMp or H-1PV at 20 PFUs/cell. (**B**) In parallel to the treatment of PBMCs, HEK293 cells cultivated in 10-cm dishes at a density of 1.5 10^6^ cells/dish were also mock-treated or infected with MVMp or H-1PV at 2 PFUs/cell. Cells (**A**, **B**) as well as supernatants (**C**) were isolated 24 hrs p.i. in order to perform Southern blot (**A**), RT-PCR (**B**), and ELISA (**C**) experiments. (**A**) DNA was extracted from hPBMCs and Southern blotting performed as described in Figure 2. (dRF, dimmer replicative form; mRF, monomeric replicative form; ssDNA, single-stranded genome). The blot shown is representative of 3 experiments which all gave similar results. (**B**) Total RNA extraction and consecutive synthesis of cDNA from hPBMC and HEK293 cultures were performed as described in Figure 1. Expression of the indicated transcripts was assessed using specific pairs of primers. Transcripts encoding the human 18S ribosomal protein were used as internal loading controls. Data shown are representative of 3 experiments that gave similar results. (**C**) Collected supernatants were centrifuged in order to discard cellular debris and analyzed by Enzyme-linked Immuno-Sorbent Assay (ELISA) for their content in IFN-α and IFN-β cytokines. Results are expressed as means+standard deviations of three independent experiments. Each presented blot is representative of 3 experiments which gave similar results.

We then determined whether the antiviral response triggered by both parvoviruses in hPBMCs requires indeed their penetration into the cells. We tested therefore whether a neuraminidase-induced hydrolysis of sialic acids present at the cell surface and known to function as receptors for both viruses, blocks the development of an antiviral response in MVMp- or H-1PV-infected PBMCs. As shown in Figure 8A, the removal of sialic acids by the glycoside hydrolase completely prevented, compared to none pre-treated cultures, the penetration of parvovirus particles into PBMCs as demonstrated by a lack of viral ssDNA genome detection in Southern blot experiments. Moreover, the induction of IFN-β transcription (Figure 8B), the production and release of this cytokine (Figure 8C) as well as its ability to activate the IFN-signaling pathway (data not shown) were all inhibited specifically in the former type of cultures. Taken together, these data indicated that the interaction of MVMp or H-1PV particles with the hPBMC surface is required for the stimulation of the IFN-mediated antiviral response.

**Figure 9:**
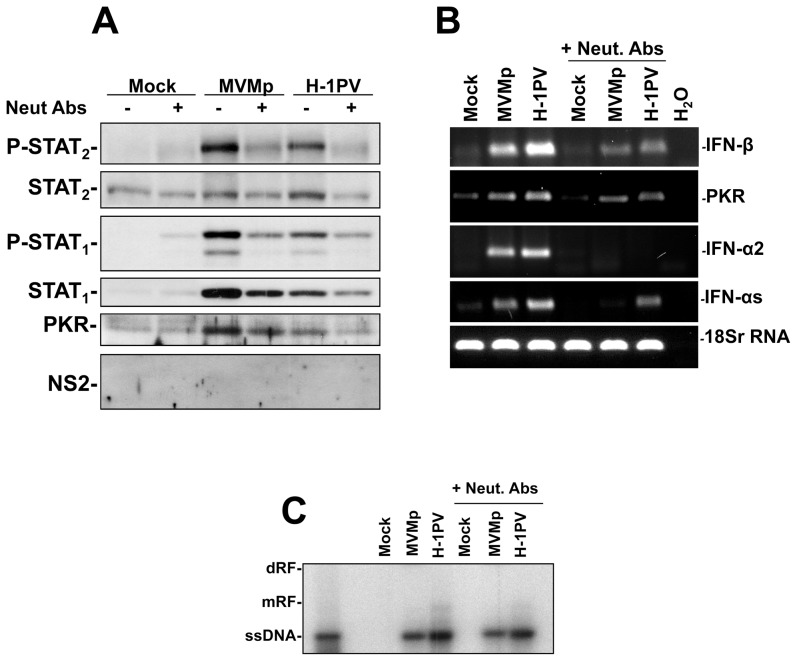
Fulfillment of the parvovirus life cycle in infected hPBMCs upon treatment with type-I IFN and IFNR2 neutralizing antibodies (Neut. Abs). (**A**, **B** and **C**) hPBMCs collected from the blood of healthy donors were distributed into 6-well plates at 1×10^7^ cells/5 ml culture medium/well. They were then treated or not with a mixture of neutralizing antibodies directed against hIFN-αs, hIFN-β, and hIFNR-2 (+Neut. Abs) at a concentration of 1 µg each/ml for 5 hrs and then mock-treated or infected with MVMp or H-1PV at 20 PFUs/cell in presence of the neutralizing antibodies. Cells (**A**, **B** and **C**) were harvested 24 hrs later by scraping in PBS and centrifuged in order to perform Western blot (**A**), RT-PCR (**B**), or Southern blot (**C**) experiments. (**A**) Cell pellets were re-suspended in complete Ripa buffer and Western blotting was performed as described in Figure 6. Actin was used as an internal loading control. Each presented blot is representative of 4 experiments which gave similar results. (**B**) Total RNA extraction and consecutive synthesis of cDNA were performed as described in Figure 1. Expression of the indicated transcripts was assessed using specific pairs of primers. Transcripts encoding the human 18S ribosomal protein were used as internal loading controls. Data shown are representative of 4 experiments which gave similar results. (**C**) Total DNA was extracted from cells and Southern blotting performed as described in Figure 2. (dRF, dimmer replicative form; mRF, monomeric replicative form; ssDNA, single-stranded genome). The blot shown is representative of 4 experiments which gave similar results.

Next, we wondered whether at least part of the parvovirus defectiveness noticed in the latter cells could be attributed to the triggered antiviral response or whether it is indeed only linked to the lack of cell proliferation. Therefore, we assessed whether a pre-treatment of hPBMC cultures with neutralizing antibodies directed against hIFN-αs, hIFN-β and their receptor (hIFNR2) restores some virus replication. Unfortunately, despite a significant reduction in the MVMp- or H-1PV-triggered activation of the IFN-signaling pathway achieved by this antibody treatment in PBMCs, as demonstrated by a weaker phosphorylation and expression of STAT_1_ and STAT_2_ proteins (Figure 9A) as well as a reduced transcription of type-I IFN (IFN-αs, -α2 and -β) and PKR genes (Figure 9B) compared to control infected cells, no replication of these viruses could be restored (Figure 9C). This finding clearly indicated that the lack of proliferation and of expression of transforming factors in hPBMCs prevent very efficiently parvovirus replication in these cells, despite the neutralization of the IFN-signaling pathway by antibodies.

### Involvement of TLR-9 in the sensing of rodent parvoviruses in hPBMCs

**Figure 10:**
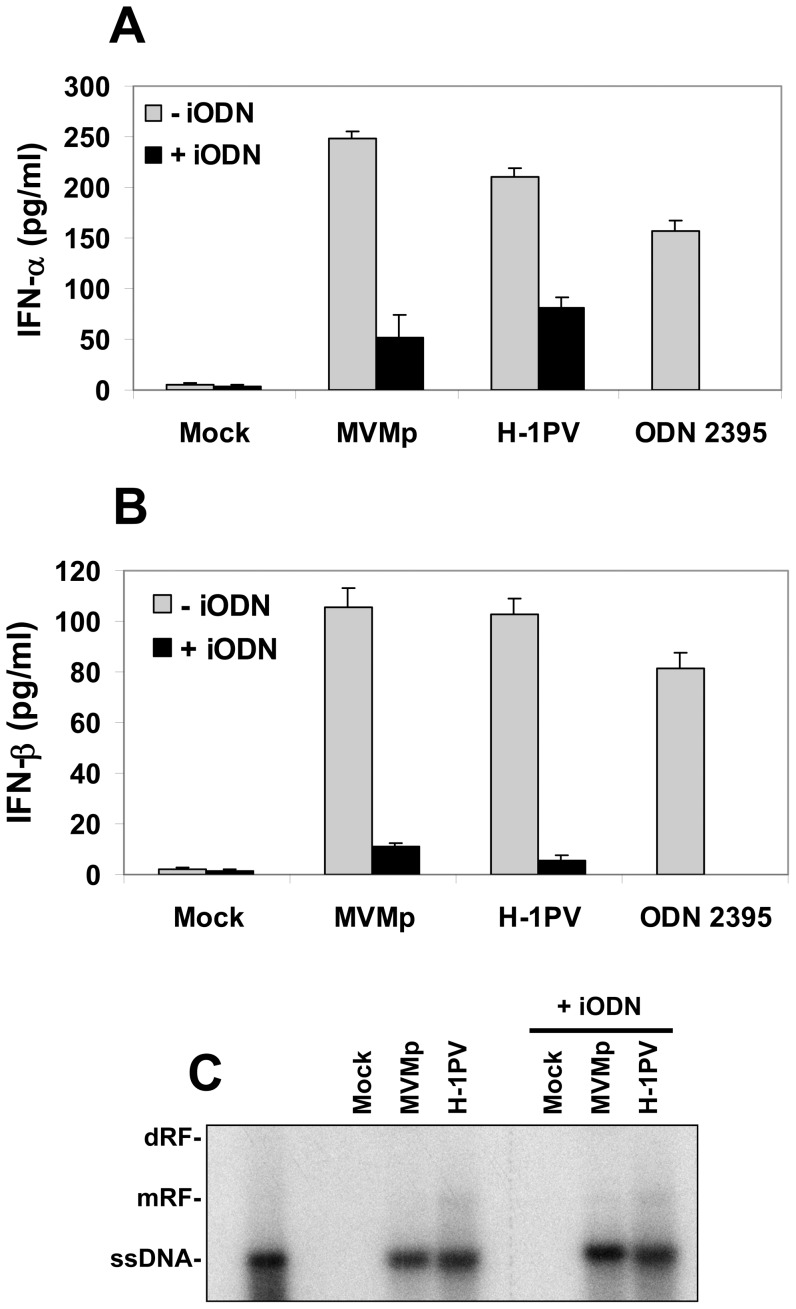
TLR-9 dependency of type-I IFN production triggered by rodent parvoviruses in infected hPBMCs. (**A**, **B** and **C**) hPBMCs were distributed into 6-well plates at 1×10^7^ cells/5 ml culture medium/well. They were immediately pre-treated, or not, for 3 hrs with the TLR-9 inhibitor ODN TTAGGG (iODN) at 2 µM and then infected or not with MVMp or H-1PV (20 PFUs/cell). In addition, some hPBMC suspensions were directly stimulated with the TLR-9 agonist ODN 2395 at 1 µM and were used as positive control experiments. Culture supernatants (**A**, **B**) as well as total DNA (**C**) were isolated 24 hrs later to perform ELISA for type-I IFNs and Southern blot experiments, respectively, as described previously in Figure 1 and 2. (**A**, **B**) Results are expressed as means+standard deviations of three independent experiments. Inhibitory effects of ODN TTAGGG on the stimulatory ODN 2395 were not assessed (ND). (**C**) (dRF, dimer replicative form; mRF, monomeric replicative form; ssDNA, single-stranded DNA genome). The blot shown is representative of 3 experiments which gave similar results.

So far we have observed that rodent parvoviruses trigger an IFN-mediated antiviral response in infected hPBMCs in absence of virus replication and protein expression suggesting that the element sensed consists in the parvoviral ssDNA genome. Toll-like receptor 9 (TLR-9) is so far the best characterized DNA-specific sensor. It triggers type-I IFN production upon the detection of unmethylated CpG motifs within the DNA sequence of invading microorganisms [47]. Given that at least two cell subsets (plasmacytoid dendritic cells (pDC) and B cells) within PBMCs possess a functional TLR-9 pathway [16], we determined whether a pre-treatment with the TLR-9 inhibitor ODN TTAGGG (iODN) [48], [49] prevents the MVMp- or H-1PV-triggered IFN production in the immune cell mixture. As shown in Figure 10, we indeed observed a partial inhibition of the parvovirus-triggered productions of IFN-α (Figure 10A) and IFN-β (Figure 10B) upon treatment of the cells with the iODN, an effect not achieved at the same concentration by a control oligodeoxynucleotide (ODN TTAGGG Control) (Figure S4). This TLR-9 inhibition could however not restore parvovirus replication (Figure 10C) in hPBMCs as expected by their lack of proliferation. Taken together, these results identified nevertheless TLR-9 as a PRR for rodent parvoviruses in hPBMCs. However, since a residual production of antiviral cytokines persisted in the infected cultures despite the TLR-9 pathway inhibition, they further suggest that additional sensors might be activated in hPBMCs upon rodent parvovirus infections.

### Assessment of a TLR-9-dependent sensing of rodent parvoviruses in transformed cells

**Figure 11:**
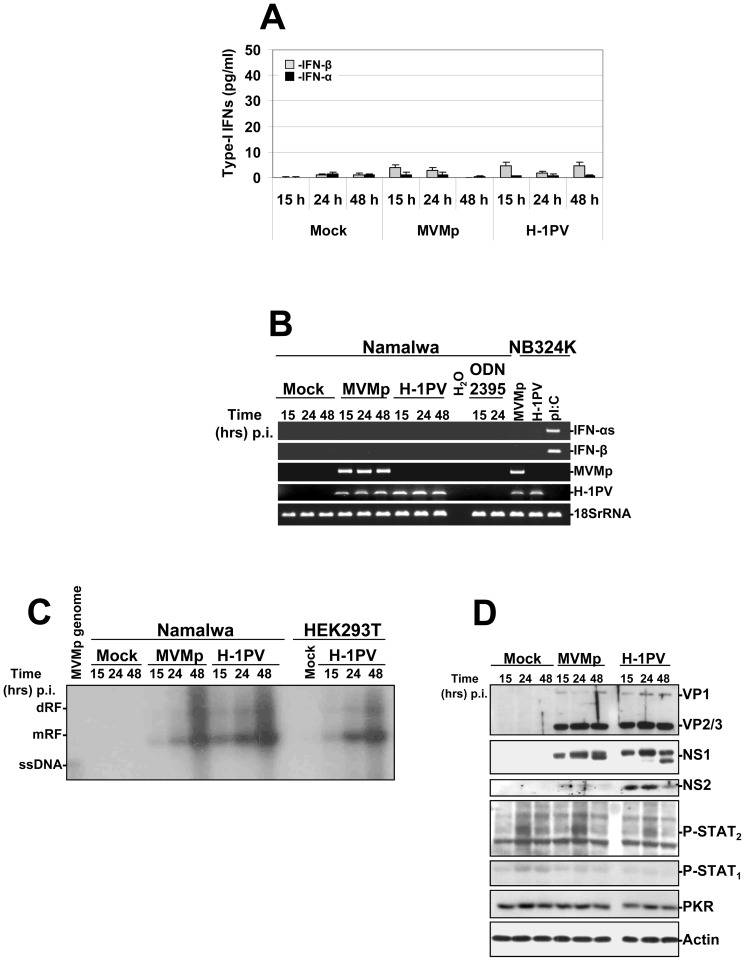
Activation of a TLR-9 dependent production of type-I IFNs in parvovirus-infected human Namalwa cells. The cells (1.10^6^ cells/well of a 24-well plate) were either infected with MVMp or H-1PV (∼80 PFUs/cell equivalent to 1×10^4^ virus genomes/cell), stimulated with the TLR-9 agonist ODN 2395 at 1 µM, or mock-treated. (**A**) At the indicated time points culture supernatants were collected from the respective wells and used to determine the quantity of type-I IFNs released in the culture medium by ELISA experiments. Results are expressed as means+standard deviations of three independent experiments. (**B**) Mock-treated, parvovirus-infected or ODN stimulated Namalwa cells were harvested at the indicated time points and total RNAs were extracted using the RNeasy kit as described previously in Figure 1. Total RNAs extracted from parvovirus-infected (24 hrs p.i. at 80 PFUs/cell) or pI:C-transfected (15 hrs post-transfection with 2 µg dsRNA/ml) NB324K cells were used as positive or negative controls. Presented data are representative of 3 experiments which all gave similar results. (**C**) Total DNA was harvested at the indicated time points from parvovirus-infected (MOI of 80 PFUs/cell) or mock-treated Namalwa or HEK293T cultures to assess by Southern blot experiments as described in Figure 2 the replication of both parvoviruses (dRF, dimmer replicative form; mRF, monomeric replicative form; ssDNA, single-stranded DNA genome). The blot shown is representative of 3 experiments which gave similar results. (**D**) Cell pellets from mock-treated or parvovirus-infected Namalwa cultures were re-suspended in complete Ripa buffer and Western blotting was performed as described in Figure 6. Actin was used as an internal loading control. Each presented blot is representative of 3 experiments which gave similar results.

The latter results prompted us to assess, since HEK293, HEK293T, NB324K and Hela cells do not express TLR-9 [50], [51], whether the Epstein-Barr Virus (EBV)-transformed Burkitt lymphoma B cells Namalwa, containing indeed a functional TLR-9 pathway [52], [53] produce and release IFNs upon MVMp or H-1PV infections performed at ∼80 PFUs/cell. However, we never detected by ELISA experiments any antiviral cytokine production in the latter infected cultures over a period of three days (Figure 11A) nor an up-regulation in the transcription of these cytokines by RT-PCRs (Figure 11B). It is interesting to note that the stimulation of the Burkitt lymphoma cell line with the TLR-9 agonist ODN 2395 at a concentration (1 µM) and for a period of time (15 to 24 hrs) previously reported to stimulate a TLR-9-dependent response (TNF and/or IL-6 production) [52], [53], also failed to trigger a type-I IFN production (Figure 11B). A deficiency in parvovirus entry into Namalwa cells can be excluded as a possible reason for the absence of cytokine production since both viruses replicated and expressed their proteins in this line as efficiently as in the parvovirus-producing cell line HEK293T (Figure 11C and D). Noteworthy, similar results were obtained (data not shown) when the latter experiments were performed with Raji cells, a second Burkitt lymphoma line highly permissive to H-1PV [54] and reported to express also elevated amounts of TLR-9 transcripts [52]. Altogether, these data indicated that parvoviruses cannot stimulate the TLR-9-dependent production of type-I IFNs in human transformed B cells. This scenario is conceivable given that normal B cells were already reported to be unable to synthesize antiviral cytokines upon stimulation of their TLR-9 pathway [55].

**Figure 12:**
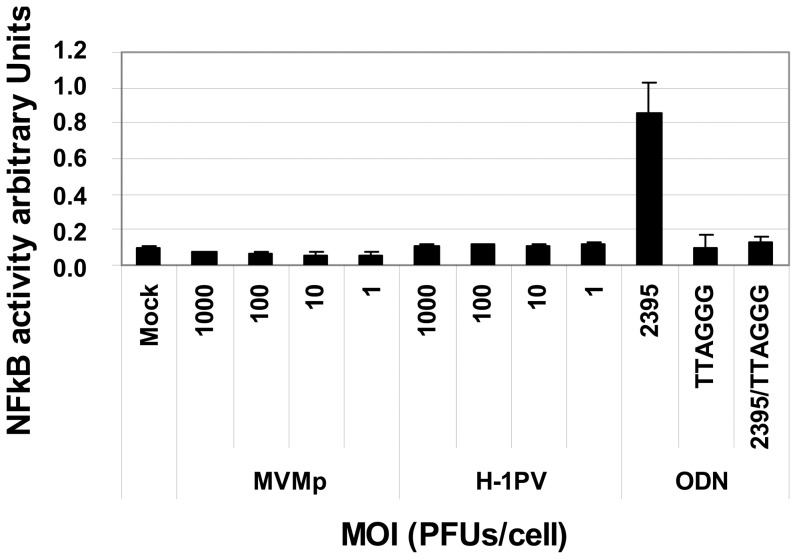
Assessment of a TLR-9-dependent activation of NF-κB in HEK-BlueTM-hTLR-9 cells upon MVMp or H-1PV infection. HEK-Blue™-hTLR-9 cells (8.10^4^ cells/well) cultivated for 24 hrs in 96-well plates following the manufacturer's instructions, were infected using increasing MOIs of MVMp or H-1PV for 24 hrs. NF-κB activity was then assessed by a colorimetric assay using a reporter system measuring the release of secreted embryonic alkaline phosphatase (SEAP) in the culture medium. Control experiments consisted in the stimulation of the latter cell line, pre-treated or not for 3 hrs with the TLR-9 inhibitor ODN TTAGGG at 2 µM, with the TLR-9 agonist ODN-2395 at 4 µM. Results are expressed as means+standard deviations of three independent experiments.

In order to determine at which level of the TLR-9 pathway the defect in parvovirus sensing is occurring in transformed cells and also to exclude a possible cell type (B cells) specificity in the latter phenotype, we tested the ability of both viruses to stimulate the transcription factor NF-κB, an early event characterizing the latter PRR-dependent response, in HEK293 cells expressing the human TLR-9 (HEK293-TLR-9^+/+^) coupled to an NF-κB reporter system. As shown in Figure 12, whereas the TLR-9 agonist ODN 2395 activated the transcription factor through an engagement of the DNA sensor, as demonstrated by a complete disappearance of the transcription factor upon a pre-treatment of the cells with the TLR-9 antagonist ODN TTAGGG, neither MVMp nor H-1PV applied at MOIs up to 1×10^3^ PFUs/cell triggered such an NF-κB activation.

Altogether, these results indicated that a defect affecting an early event in the TLR-9 recognition of parvovirus genomes, like an absence of exposure of the viral ssDNA to the sensor in endosomes, prevents in transformed cells the TLR-9 sensing of these infectious agents.

## Discussion

We have previously shown that MVMp triggers an antiviral response *via* the production of type-I IFNs in normal mouse fibroblasts but not in transformed counterparts. This defense mechanism hampers the replication of the virus in the former but not in the latter type of cells regulating thereby the virus tropism (oncotropism) [29]. The present study provides evidences demonstrating for the first time to our knowledge that similar antiviral processes are also evoked by MVMp and H-1PV in normal but not in transformed/tumor cells of human origin. Indeed, we show that upon rodent parvovirus infection, human peripheral blood mononuclear cells (hPBMCs) produce and release both IFN-α and -β molecules and show hallmarks of activation of an antiviral response whereas human transformed/tumor cells, being or not of immune origin, never showed such effects upon MVMp- or H-1PV infection. PBMCs were used here as normal cellular model since this cell mixture is composed among others of plasmacytoid dendritic cells (pDCs) known to represent the almost unique cell type in humans able to produce a large amount of type-I IFNs upon its infection [56], [57], [58] and also because the whole repertoire of so far known PRRs is expressed in a functional state in this mixed cell population.

Our work also points to TLR-9 as the PRR sensing rodent parvovirus infections in hPBMCs. Indeed, this receptor is a viral DNA sensor expressed in endosomes [11] and able to trigger specifically in immune cells a type-I IFN production upon its activation [59], [60]. This finding suggests that the parvovirus genome, although being described for MVMp to contain less CpG motifs than Adeno-associated viruses (AAVs) [61] and for H-1PV to be a weak activator of a TLR-9 response [62], [63], is nevertheless composed of sufficient motifs to be recognized as a PAMP. These results are in agreement with two previous reports showing that AAV2 as well as KRV, both being members of the *Parvoviridae* family, are sensed by TLR-9 in human and/or rodent immune cells [33], [64]. In hPBMCs, only pDCs and B cells are known to possess a functional TLR-9 pathway [16], however, while the former cell type indeed produces and releases massive amounts of type-I IFNs upon a TLR-9 activation, the latter rather synthesizes inflammatory cytokines like TNFα and IL-6 upon engagement of the DNA sensor [11], [55]. Given in addition that TLR-9 was demonstrated to be essential in pDCs for the recognition of unmethylated CpG oligodeoxynucleotides [16], our findings clearly point to pDCs as the cell type contributing the most to the production of type-I IFNs in rodent parvovirus-infected hPBMCs.

Given the latter scenario holds true, it could imply that normal non-immune cells because of an absence of TLR-9 expression or an intrinsic deficiency in its downstream pathway should be unable to develop a type-I IFN-mediated antiviral response upon parvovirus infection. However, in such type of cells additional steps of the parvovirus life cycle are susceptible to be achieved compared to PBMCs, offering thereby the possibility for other antiviral factors and sensors to detect the parvovirus presence. This assumption is however in contradiction with a recent report claiming that AAV2 can trigger an IFN-mediated antiviral response in normal human lung fibroblasts (HLFs) through a TLR-9 engagement [65]. However, since in this work no data were provided demonstrating that a TLR-9 activation, for instance by a synthetic oligodeoxynucleotide, is indeed able to trigger an IFN production or that a TLR-9 inhibitor prevents the observed AAV-triggered IFN release, it remains possible that another PRR may have contributed to the latter antiviral response in HLFs.

The existence in non-immune cells of parvovirus sensors different from TLR-9 is rather appealing. It is moreover supported by observations made in our previous study showing that an IFN production is triggered by MVMp in infected mouse embryonic fibroblasts [29] reported to be deficient for antiviral cytokine production upon stimulation of their TLR-9 pathway by a double-stranded CpG DNA [66].

Interestingly, based on our data it could also be proposed that an additional parvoviral PRR, besides TLR-9, is also triggered in infected hPBMCs. Indeed, since the TLR-9 inhibitor ODN TTAGGG [48], [49] fully blocks the TLR-9-mediated activation of NFκB in HEK293-hTLR9^+/+^ cells, but only partially inhibits the parvovirus-triggered IFN production in hPBMCs, it suggests that another factor (PRR) might contribute, besides TLR-9, to the latter cytokine synthesis. This potential additional sensor belongs most likely to the family of DNA sensors [67], [68] since both MVMp and H-1PV infections remained defective over the period of time tested, in agreement with previous data reported by our group [30] but in contradiction with others that noticed some H-1PV NS1 polypeptide expression in infected PBMCs [69]. However, it cannot be excluded so far that some discreet parvoviral RNAs, different from those tested in our RT-PCR experiments (encoded by the NS/VP genes), are synthesized in infected peripheral blood mononuclear cells and that these nucleic acids could activate an RNA-sensing PRR. It could consist for instance in TLR-7, another member of the Toll-like Receptor family, known to sense viral ssRNAs, but also to indirectly contribute to the detection of CpG motifs by TLR-9 [16].

The absence of IFN production and antiviral response triggered by both rodent parvoviruses in the human cell lines HEK293, HEK293T, NB324K and Hela can be obviously explained by an absence of TLR-9 expression [50]. However, in transformed immune cells that express this PRR like in the Namalwa or Raji line (EBV-transformed B cells) [52], it could be postulated that their TLR-9 pathway is intrinsically unable to trigger an antiviral cytokine production as previously shown for normal B cells [55]. It could also be proposed that EBV factors impair the TLR-9-dependent IFN production in the Burkitt lymphoma cells as described in several studies [70], [71], or that the efficient multiplication observed for both rodent parvoviruses in the B cell lines [54] allows the development of an evasion mechanism inhibiting at a very early step (before NF-κB activation) the TLR-9 pathway. This possibility sounds to our opinion not far-fetched in particular since both TLR-7- and TLR-9-dependent immune responses were recently shown to dependent on many co-factors [72] representing as many possibilities given to a virus in order to intervene and block the fulfillment of both pathways. Finally, a weak, bad, or deficient exposure to TLR-9 of the parvovirus ssDNA genome within endosomes of transformed cells could also be proposed to explain the absence of IFN production triggered by MVMp and H-1PV in Namalwa and Raji cells.

Unfortunately, our data do not provide sufficient evidences in order to claim that the antiviral response triggered by MVMp and H-1PV in hPBMCs contributes to the defectiveness of these agents nor to their oncotropic behavior towards human cells [73] as it was shown for several other viruses [9] and for MVMp in mouse cells [29]. Indeed, our attempts to restore a productive parvovirus life cycle in infected hPBMC cultures either by inhibiting the stimulation of the antiviral response (Jak/STAT pathway) using neutralizing antibodies or by preventing the synthesis of antiviral cytokines using a TLR-9 antagonist, failed. The fact that hPBMCs do not proliferate in culture and lack oncogenic transformation most likely accounts for the absence of parvovirus replication in these cells even in the presence of a treatment inhibiting antiviral mechanisms. In contrast, an entry defect can a priori be excluded as a potential explanation since we showed that MVMp and H-1PV bind and enter efficiently into PBMCs. However, an insufficient inhibition of the IFN-signaling (Jak/STAT) or the IFN-production pathway achieved by the neutralizing antibodies as well as by the TLR-9 inhibitor could also be proposed to account, at least to some extent, to the lack of parvovirus replication in these cells.

Altogether, this study provides for the first time evidences demonstrating that normal human immune cells sense MVMp and H-1PV infections in a TLR-9-dependent manner, a feature that leads to type-I IFN production and to the downstream development of an antiviral response. Since antiviral cytokines are known to regulate the innate as well as the adaptive arm of the immune system [74], their production by normal immune cells, like pDCs, upon rodent parvovirus infections could contribute both to the regulation of the oncotropism of these agents and to the stimulation *in vivo* of an anticancer immune response.

## Supporting Information

Figure S1 **Sensitivity of human cell lines to the antiviral action of recombinant human IFN-β.** HEK293, HEK293T, NB324K and Hela cultures were pre-incubated or not for 16 hrs with 500 IU/ml of recombinant human IFN-β (rhIFN-β). Both type of cultures were then mock-treated or infected with MVMp or H-1PV at 5 PFUs/cell. The monolayers were harvested 48 hrs later and total DNA was extracted using a modified Hirt extraction method as described in Materials and Methods. DNA samples were then digested with proteinase K and 2 µg of total DNA from each sample was then subjected to eletrophoresis through a 0.8% agarose gel and further transferred by capillarity on a Hybond-N membrane. Expression of DNA intermediates was investigated using a mixture of radiolabeled DNA probes corresponding to the E*co* RI-E*co* RV and H*ind* III-H*ind* III fragments of the viral NS genes from MVMp and H-1PV, respectively. Assessment of the migration of MVMp isolated genomes (0.08 µg) was used in each blot as migration control of the different DNA intermediates; mRF, monomeric replicative form; dRF, dimmer replicative form; ssDNA, single-stranded genome. The blot shown is representative of 3 experiments all of which gave similar results.
(TIF)

Figure S2 **Production and release of IFN-β from human transformed or tumor cells upon MVMp or H-1PV infection.** HEK293, HEK293T, NB324K and Hela cultures were mock-treated for 48 hrs, parvovirus-infected (5 PFUs/cell) for 48 hrs, transfected with 2 µg/ml of pI:C for 15 hrs or infected with NDV at 6 HU/10^6^ cells for 15 hrs. The culture media were then collected, centrifuged to discard cellular debris, and analyzed by Enzyme-linked Immuno-Sorbent Assay (ELISA) for their content in human IFN-β. Each result is represented as mean+standard deviation of three independent experiments.
(TIF)

Figure S3 **Activation of the IFN-signaling (Jak/STAT) pathway in human cell lines upon rodent parvovirus infections.** (**A**) HEK293, HEK293T, (**B**) NB324K and (**C**) Hela cells were mock-treated or infected with rodent parvoviruses MVMp or H-1PV at 5 PFUs/cell. In addition, HEK-293 and HEK-293T cultures (**A**) were also transfected with pI:C at 2 µg/ml or infected with NDV (6 HU/10^6^ cells) while NB324K (**B**) and Hela (**C**) cells were only additionally infected with NDV at the same MOI as in HEK cultures. At the time point indicated in each figure for infected cultures, at 24 hrs for mock-treated cells and after 15 hrs for pI:C-transfected or NDV-infected monolayers, cultures were harvested by scraping in PBS and centrifuged. Cell pellets were then re-suspended in complete Ripa buffer supplemented with phosphatase and protease inhibitors. Total proteins were extracted from each sample as described in Materials and Methods. Seventy µg total proteins per sample were then subjected to bipartite 8/10% SDS-PAGE, transferred onto membranes, and probed with antibodies specific for phosphorylated and total isoforms of STAT_1_ and STAT_2_. as well as with an antibody specific to PKR or NS1 (SP8). Actin was used as an internal loading control. Each presented blot is representative of 3 additional which gave similar results.
(TIF)

Figure S4 **Effect of the oligodeoxynucleotide ODN TTAGGG Control on the TLR-9-mediated production and release of type-I IFNs from parvovirus-infected hPBMCs.** hPBMCs were distributed into 6-well plates at 1×10^7^ cells/5 ml culture medium/well. They were immediately pre-treated, or not, for 3 hrs with the TLR-9 inhibitor ODN TTAGGG or its control homolog ODN TTAGGG Control at 2 µM and then infected, or not, for 24 hrs with MVMp or H-1PV (20 PFUs/cell). Culture supernatants were then harvested and ELISA for type-I IFNs was performed following the manufacturer's instructions. Results are expressed as means+standard deviations of three independent experiments performed in duplicate.
(TIF)
