# Supplementary figures and images for: TLR-9 Contributes to the Antiviral Innate Immune Sensing of Rodent Parvoviruses MVMp and H-1PV by Normal Human Immune Cells
Source: PLoS One. 2013 Jan 29;8(1):e55086. doi: 10.1371/journal.pone.0055086 (PMC3558501; doi:10.1371/journal.pone.0055086)

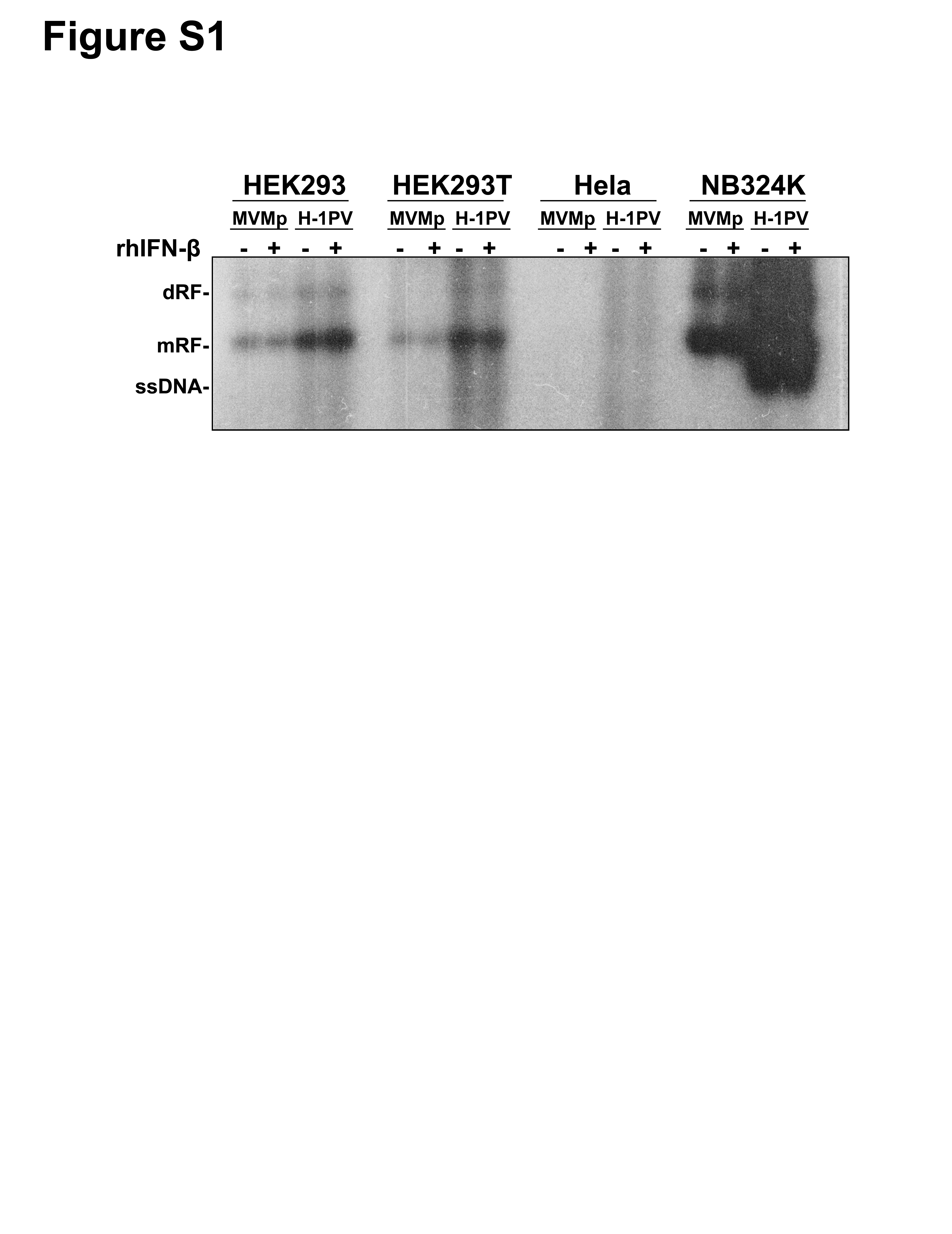

Supplement: Figure S1 — Sensitivity of human cell lines to the antiviral action of recombinant human IFN-β. HEK293, HEK293T, NB324K and Hela cultures were pre-incubated or not for 16 hrs with 500 IU/ml of recombinant human IFN-β (rhIFN-β). Both type of cultures were then mock-treated or infected with MVMp or H-1PV at 5 PFUs/cell. The monolayers were harvested 48 hrs later and total DNA was extracted using a modified Hirt extraction method as described in Materials and Methods. DNA samples were then digested with proteinase K and 2 µg of total DNA from each sample was then subjected to eletrophoresis through a 0.8% agarose gel and further transferred by capillarity on a Hybond-N membrane. Expression of DNA intermediates was investigated using a mixture of radiolabeled DNA probes corresponding to the Eco RI-Eco RV and Hind III-Hind III fragments of the viral NS genes from MVMp and H-1PV, respectively. Assessment of the migration of MVMp isolated genomes (0.08 µg) was used in each blot as migration control of the different DNA intermediates; mRF, monomeric replicative form; dRF, dimmer replicative form; ssDNA, single-stranded genome. The blot shown is representative of 3 experiments all of which gave similar results. (TIF) [file pone.0055086.s001.tif]

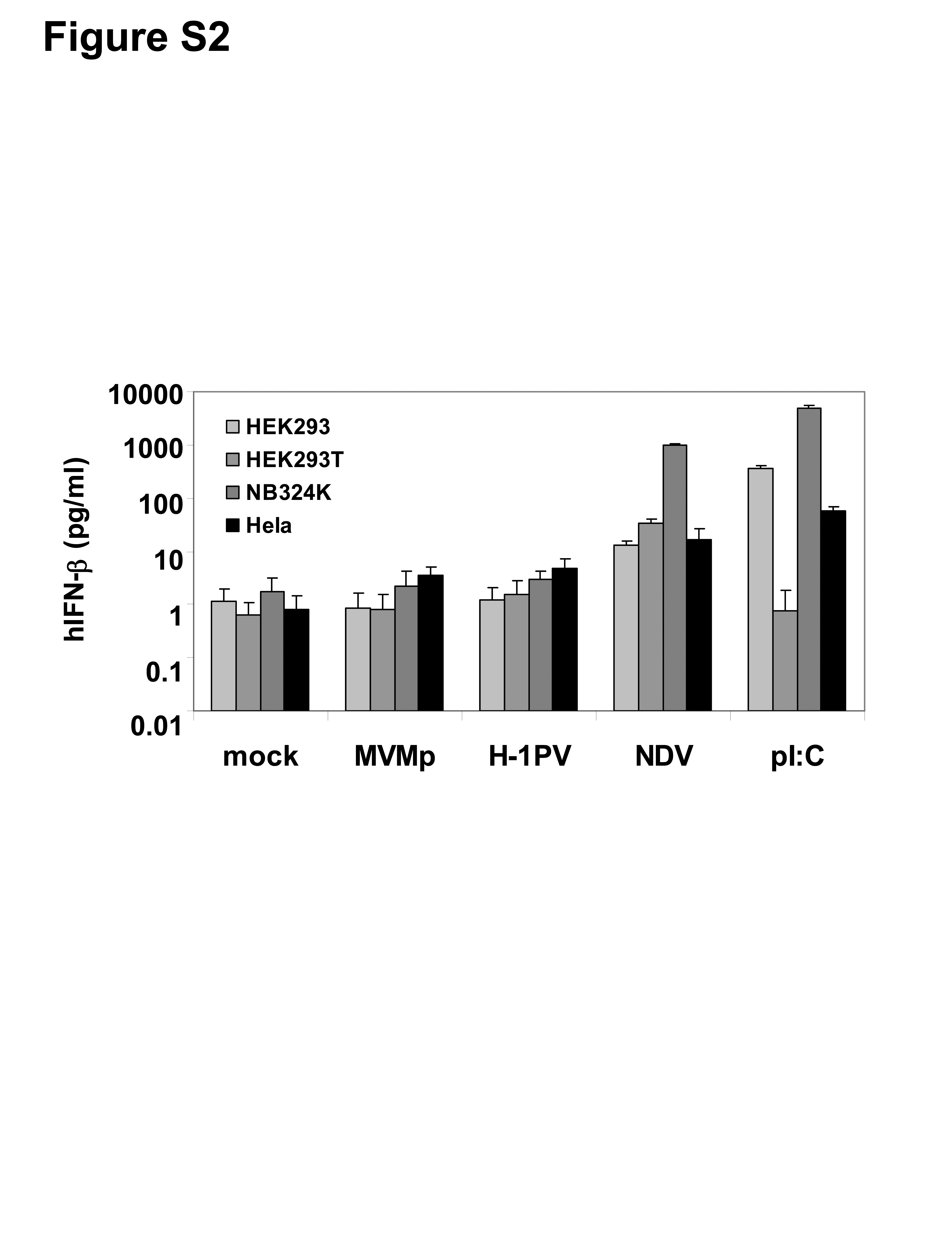

Supplement: Figure S2 — Production and release of IFN-β from human transformed or tumor cells upon MVMp or H-1PV infection. HEK293, HEK293T, NB324K and Hela cultures were mock-treated for 48 hrs, parvovirus-infected (5 PFUs/cell) for 48 hrs, transfected with 2 µg/ml of pI:C for 15 hrs or infected with NDV at 6 HU/106 cells for 15 hrs. The culture media were then collected, centrifuged to discard cellular debris, and analyzed by Enzyme-linked Immuno-Sorbent Assay (ELISA) for their content in human IFN-β. Each result is represented as mean+standard deviation of three independent experiments. (TIF) [file pone.0055086.s002.tif]

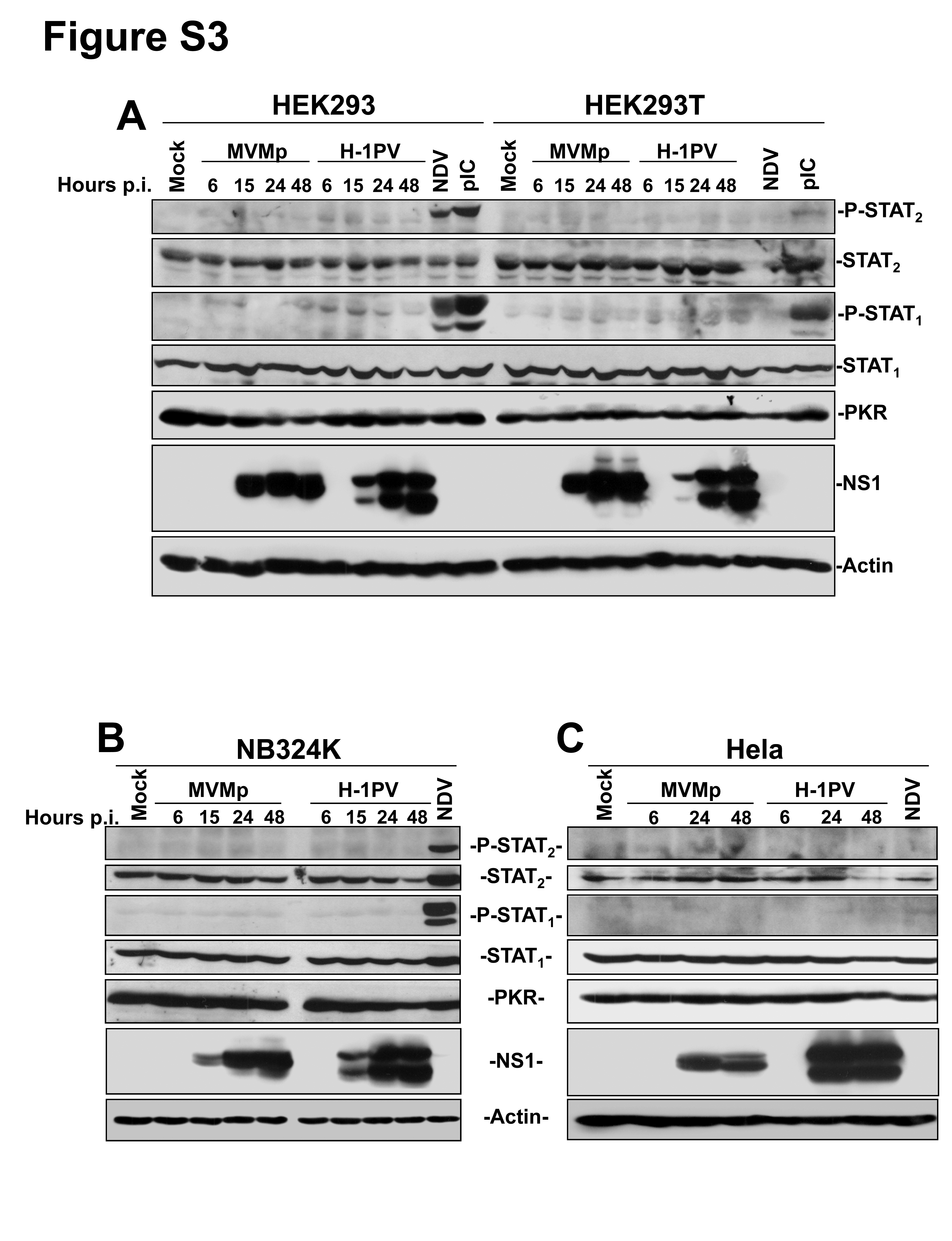

Supplement: Figure S3 — Activation of the IFN-signaling (Jak/STAT) pathway in human cell lines upon rodent parvovirus infections. (A) HEK293, HEK293T, (B) NB324K and (C) Hela cells were mock-treated or infected with rodent parvoviruses MVMp or H-1PV at 5 PFUs/cell. In addition, HEK-293 and HEK-293T cultures (A) were also transfected with pI:C at 2 µg/ml or infected with NDV (6 HU/106 cells) while NB324K (B) and Hela (C) cells were only additionally infected with NDV at the same MOI as in HEK cultures. At the time point indicated in each figure for infected cultures, at 24 hrs for mock-treated cells and after 15 hrs for pI:C-transfected or NDV-infected monolayers, cultures were harvested by scraping in PBS and centrifuged. Cell pellets were then re-suspended in complete Ripa buffer supplemented with phosphatase and protease inhibitors. Total proteins were extracted from each sample as described in Materials and Methods. Seventy µg total proteins per sample were then subjected to bipartite 8/10% SDS-PAGE, transferred onto membranes, and probed with antibodies specific for phosphorylated and total isoforms of STAT1 and STAT2. as well as with an antibody specific to PKR or NS1 (SP8). Actin was used as an internal loading control. Each presented blot is representative of 3 additional which gave similar results. (TIF) [file pone.0055086.s003.tif]

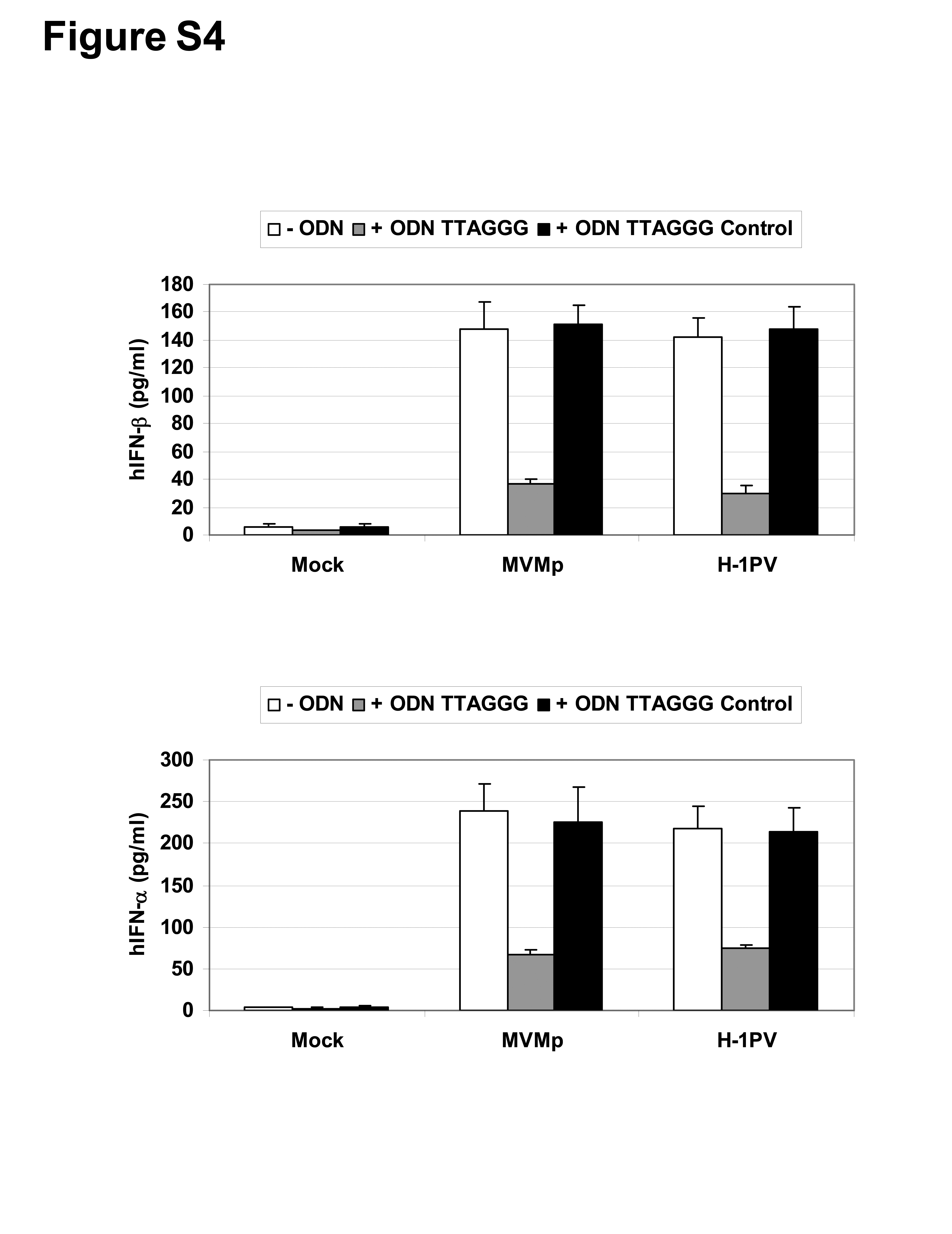

Supplement: Figure S4 — Effect of the oligodeoxynucleotide ODN TTAGGG Control on the TLR-9-mediated production and release of type-I IFNs from parvovirus-infected hPBMCs. hPBMCs were distributed into 6-well plates at 1×10^7^ cells/5 ml culture medium/well. They were immediately pre-treated, or not, for 3 hrs with the TLR-9 inhibitor ODN TTAGGG or its control homolog ODN TTAGGG Control at 2 µM and then infected, or not, for 24 hrs with MVMp or H-1PV (20 PFUs/cell). Culture supernatants were then harvested and ELISA for type-I IFNs was performed following the manufacturer's instructions. Results are expressed as means+standard deviations of three independent experiments performed in duplicate. (TIF) [file pone.0055086.s004.tif]
